# Crystal-structure studies of 4-phenyl­piperazin-1-ium 4-eth­oxy­benzoate monohydrate, 4-phenyl­piperazin-1-ium 4-meth­oxy­benzoate monohydrate, 4-phenyl­piperazin-1-ium 4-methyl­benzoate monohydrate and 4-phenyl­piperazin-1-ium tri­fluoro­acetate 0.12-hydrate

**DOI:** 10.1107/S2056989022006004

**Published:** 2022-06-10

**Authors:** Ninganayaka Mahesha, Haruvegowda Kiran Kumar, Mehmet Akkurt, Hemmige S. Yathirajan, Sabine Foro, Mohammed S. M. Abdelbaky, Santiago Garcia-Granda

**Affiliations:** a Department of Studies in Chemistry, University of Mysore, Manasagangotri, Mysore-570 006, India; b Department of Physics, Faculty of Sciences, Erciyes University, 38039 Kayseri, Turkey; c Institute of Materials Science, Darmstadt University of Technology, Alarich-Weiss-Strasse 2, D-64287 Darmstadt, Germany; d Department of Physical and Analytical Chemistry, Faculty of Chemistry, Oviedo University-CINN, Oviedo 33006, Spain

**Keywords:** crystal structure, hydrogen bonding, piperazine, biological activity

## Abstract

In this study, four new piperazinium salts, namely, 4-phenyl­piperazin-1-ium 4-eth­oxy­benzoate monohydrate, C_9_H_9_O_3_·C_10_H_15_N_2_·H_2_O (**I**); 4-phenyl­piperazin-1-ium 4-meth­oxy­benzoate monohydrate, C_10_H_15_N_2_·C_8_H_7_O_3_·H_2_O (**II**); 4-phenyl­piperazin-1-ium 4-methyl­benzoate monohydrate, C_10_H_15_N_2_·C_8_H_7_O_2_·H_2_O (**III**); and 4-phenyl­piperazin-1-ium tri­fluoro­acetate 0.12 hydrate, C_10_H_15_N_2_·C_2_F_3_O_2_·0.12H_2_O (**IV**), have been synthesized. The single-crystal structures of these compounds reveal that all of them crystallize in the triclinic *P*  space group and the crystal packing of (**I**)–(**III**) is built up of ribbons formed by a combination of hydrogen bonds of type N—H⋯O, O—H⋯O and other weak inter­actions of type C—H⋯O and C—H⋯π, leading to a three-dimensional network. In the crystal of (**IV**), the cations and the anions are connected by C—H⋯O, N—H⋯O and C—H⋯F hydrogen bonds and by C—H⋯π inter­actions, forming sheets which in turn inter­act to maintain the crystal structure by linking through the oxygen atoms of water mol­ecules and van der Waals inter­actions, giving the whole structure.

## Abstract 2

Four novel piperazinium salts are reported, and based on 1-phenyl­piperazinium as a common cation in the asymmetric units that include additionally water mol­ecules, and different *P*-substituent benzoate anions or a tri­fluoro­acetate anion. They are hydrated and there are three crystallized as 1:1 salts while the fourth is a 2:2 salt. Their crystal packing depends on strong ribbons or sheets stabilized by hydrogen bonds of type N—H⋯O and O—H⋯O and other inter­actions as C—H⋯O, C—H⋯π and C—H⋯F in the tri­fluoro­acetate-based one.

## 1. Chemical context

Piperazines are among the most important building blocks in today’s drug discovery efforts and are found in biologically active compounds across a number of different therapeutic areas (Brockunier *et al.*, 2004 ▸; Bogatcheva *et al.*, 2006 ▸). For a review on the current pharmacological and toxicological information for piperazine derivative, see Elliott (2011 ▸). Various pharmacological properties of phenyl­piperazines and their derivatives have been discussed by several authors (Cohen *et al.*, 1982 ▸; Conrado *et al.*, 2010 ▸; Neves *et al.*, 2003 ▸; Hanano *et al.*, 2000 ▸). The design and synthesis of phenyl­piperazine derivatives as potent anti­cancer agents for prostate cancer have been described (Demirci *et al.*, 2019 ▸). Many pharmaceutical compounds are derived from 1-phenyl­piperazine, *viz.*, oxypertine, trazodone, nefazodone, *etc*.

The crystal structures of 2-(4-methyl-2-phenyl­piperazin-4-ium-1-yl)pyridine-3-carboxyl­ate dehydrate (Li *et al.*, 2008 ▸), 1-chloro-2-(4-phenyl­piperazin-1-yl)-ethanone (Xu & Jing, 2009 ▸), 4-phenyl­piperazin-1-ium di­hydrogen phosphate (Essid *et al.*, 2010 ▸) and 1-phenyl­piperazine-1,4-diium bis­(hydrogen sulfate) (Marouani *et al.*, 2010 ▸) have been reported, as have those of 4-phenyl­piperazin-1-ium 6-chloro-5-ethyl-2,4-dioxopyrimidin-1-ide and 4-phenyl­piperazin-1-ium 6-chloro-5-isopropyl-2,4-dioxopyrimidin-1-ide (Al-Alshaikh *et al.*, 2015 ▸). We have reported the crystal structures of some salts of 4-meth­oxy­phenyl­piperazine (Kiran Kumar *et al.*, 2019*a* ▸), six 1-aroyl-4-(4-meth­oxy­phen­yl)piperazines (Kiran Kumar *et al.*, 2019*b* ▸), 2-meth­oxy­phenyl­piperazine (Harish Chinthal *et al.*, 2020 ▸) and the recreational drug *N*-(4-meth­oxy­phen­yl)piperazine (MeOPP) and three of its salts (Kiran Kumar *et al.*, 2020*a* ▸). 
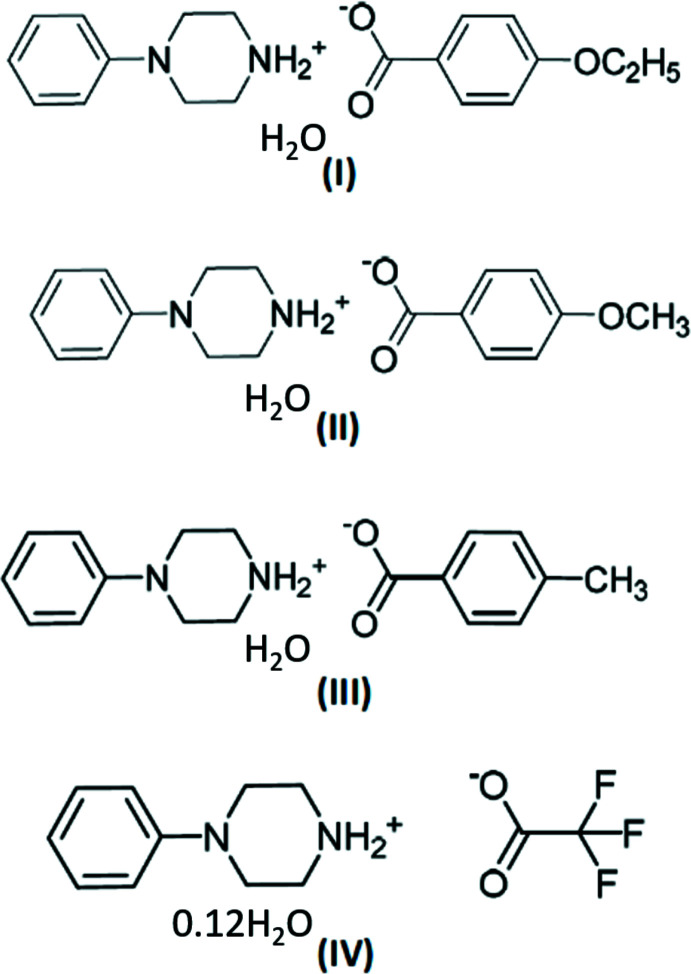


In view of the importance of piperazines in general and the use of 1-phenyl­piperazine in particular, the present paper reports the crystal structure studies of some salts of 1-phenyl­piperazine with organic acids *viz*., 4-phenyl­piperazin-1-ium 4-eth­oxy­benzoate monohydrate, C_9_H_9_O_3_·C_10_H_15_N_2_·H_2_O (**I**); 4-phenyl­piperazin-1-ium 4-meth­oxy­benzoate monohydrate, C_10_H_15_N_2_·C_8_H_7_O_3_·H_2_O (**II**); 4-phenyl­piperazin-1-ium 4-methyl­benzoate monohydrate, C_10_H_15_N_2_·C_8_H_7_O_2_·H_2_O (**III**); and 4-phenyl­piperazin-1-ium tri­fluoro­acetate 0.12 hydrate C_10_H_15_N_2_·C_2_F_3_O_2_·0.12H_2_O (**IV**).

## 2. Structural commentary

**Figure 1:**
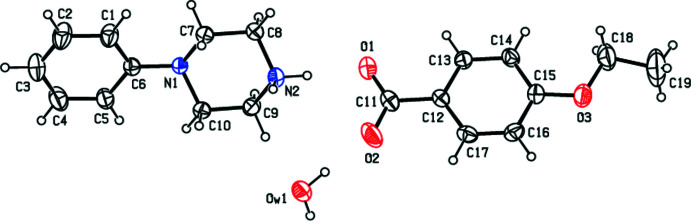
The independent components of compound (**I**) showing the atom-labelling scheme. Displacement ellipsoids are drawn at the 30% probability level.

**Figure 2:**
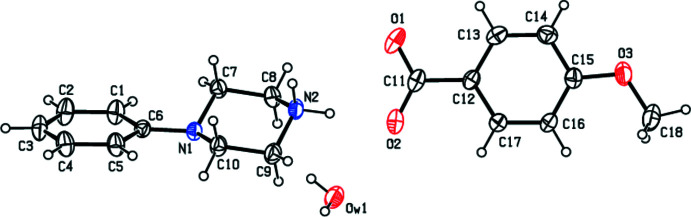
The independent components of compound (**II**) showing the atom-labelling scheme. Displacement ellipsoids are drawn at the 30% probability level.

**Figure 3:**
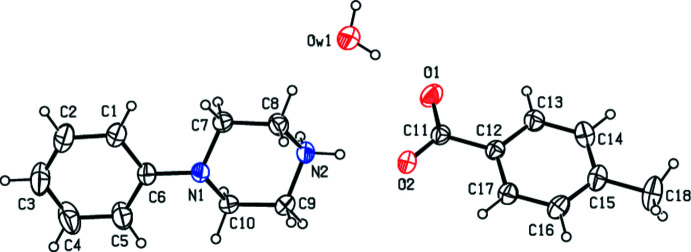
The independent components of compound (**III**) showing the atom-labelling scheme. Displacement ellipsoids are drawn at the 30% probability level.

**Figure 4:**
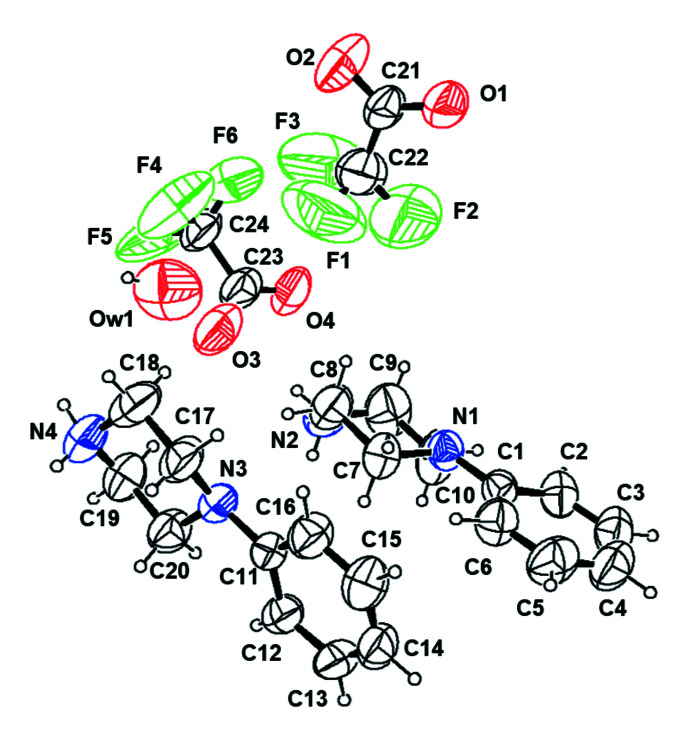
The independent components of compound (**IV**) showing the atom-labelling scheme. Displacement ellipsoids are drawn at the 30% probability level. (Atom splitting is omitted for clarity.)

The asymmetric unit of the compound (**I**), (Fig. 1 ▸), consists of a 4-phenyl­piperazin-1-ium cation, a 4-eth­oxy­benzoate anion and one water mol­ecule. The aromatic ring of the cation is essentially planar while the protonated piperazine ring adopts a chair conformation, with puckering parameters (Cremer & Pople, 1975 ▸) *Q*_T_ = 0.553 (2) Å, θ = 175.0 (2)° and φ = 15 (3)°. In compound (**II**) the asymmetric unit (Fig. 2 ▸) comprises a 4-phenyl­piperazin-1-ium cation, a 4-meth­oxy­benzoate anion and one water mol­ecule. The aromatic ring of the cation is essentially planar while the protonated piperazine ring adopts a chair conformation, with puckering parameters *Q*_T_ = 0.5614 (18) Å, θ = 175.89 (17)° and φ = 346 (3)°. Compound (**III**) presents an asymmetric unit (Fig. 3 ▸) composed of a 4-phenyl­piperazin-1-ium cation, a 4-methyl­benzoate anion and one water mol­ecule. The aromatic ring of the cation is essentially planar but the protonated piperazine ring adopts a distorted chair conformation, with puckering parameters *Q*_T_ = 0.5486 (19) Å, θ = 9.38 (19)° and φ = 167.9 (13)°. On the other hand, the asymmetric unit of (**IV**) (Fig. 4 ▸) contains two 4-phenyl­piperazin-1-ium cations (*A*1 with N1, *A*2 with N3) and two tri­fluoro­acetate anions (*B*1 with F1, *B*2 with F4) and a 0.12 occupancy water molecule. The aromatic rings of the cations (*A*1, *A*2) are essentially planar while the protonated piperazine rings adopt a chair conformation for cation *A*1, with puckering parameters (Cremer & Pople, 1975 ▸) *Q*_T_ = 0.552 (4) Å, θ = 0.0 (4)° and φ = 207 (14)°, and a distorted chair conformation for the cation *A*2, with puckering parameters *Q*_T_ = 0.559 (5) Å, θ = 6.6 (4)° and φ = 168 (4)°.

## 3. Supra­molecular features

**Figure 5:**
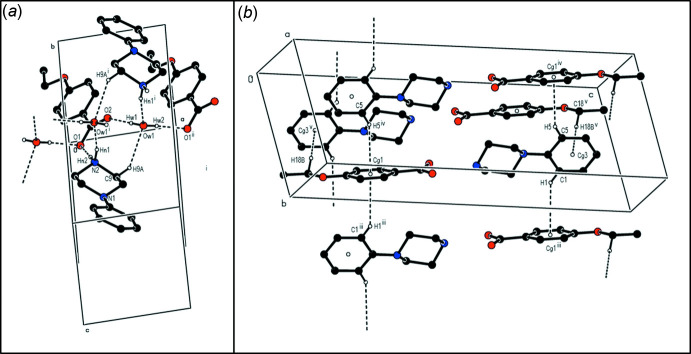
Parts of the crystal structure of compound (**I**) showing (*a*) the formation of a cyclic hydrogen-bonded 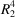 (10) aggregate and (*b*) a general view of C—H⋯π inter­actions parallel to [100]. Hydrogen bonds and C—H⋯π inter­actions are drawn as dashed lines.

In the crystal structure of (**I**), the cation pairs are connected across two water mol­ecules by C—H⋯O and N—H⋯O hydrogen bonds, forming an 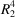 (10) ring motif in which the anions and cations are linked through the water mol­ecules by O—H⋯O and N—H⋯O hydrogen bonds, forming ribbons along the *a*-axis direction (Table 1 ▸, Fig. 5 ▸ *a*). In addition, a set of C—H⋯π inter­actions, through the benzene rings of the anions and the cations, connect the mol­ecules together in ribbons along the *a*-axis direction (Table 1 ▸, Fig. 5 ▸ *b*). The C—H⋯O, N—H⋯O, O—H⋯O hydrogen bonds and C—H⋯π inter­actions together form a three-dimensional network, contributing to the stabilization of the crystal structure.

**Figure 6:**
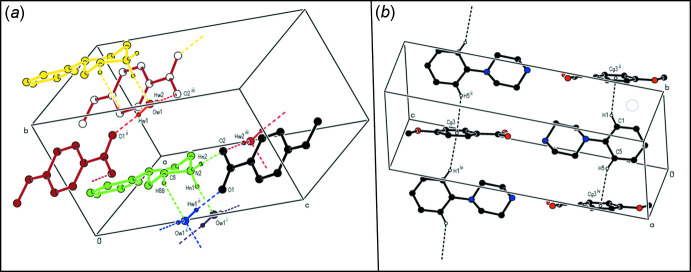
Parts of the crystal structure of compound (**II**) showing (*a*) the formation of hydrogen-bonded ribbons parallel to [010] and (*b*) a general view of the C—H⋯π inter­actions parallel to [010]. Hydrogen bonds and C—H⋯π inter­actions are drawn as dashed lines.

In the crystal structure of (**II**), the cations, the anions and the water mol­ecules are connected by C—H⋯O, N—H⋯O and O—H⋯O hydrogen bonds, forming ribbons along the *a*-axis direction (Table 2 ▸, Fig. 6 ▸ *a*). Furthermore, the cations inter­act *via* C—-H⋯π inter­actions through the benzene ring of the anion, forming ribbons along the *b*-axis direction (Table 2 ▸, Fig. 6 ▸ *b*). The C—H⋯O, N—H⋯O, O—H⋯O hydrogen bonds and C—H⋯π inter­actions together form a three-dimensional network, contributing to the stabilization of the crystal structure.

**Figure 7:**
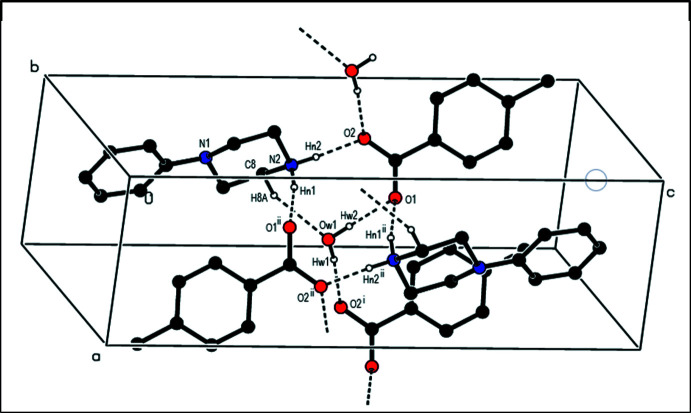
Part of the crystal structure of compound (**III**) showing the formation of a hydrogen-bonded chain of rings parallel to [001]. Hydrogen bonds are drawn as dashed lines.

In the crystal structure of (**III**), the cations, the anions and the water mol­ecules are connected by C—H⋯O, N—H⋯O and O—H⋯O hydrogen bonds, forming ribbons along the *a*-axis direction (Table 3 ▸, Fig. 7 ▸). There are no C—H⋯π inter­actions or π-π stacking inter­actions. The crystal structure is stabilized by C—H⋯O, N—H⋯O, O—H⋯O hydrogen bonds and van der Waals inter­actions between the ribbons, which run along the *a*-axis direction.

**Figure 8:**
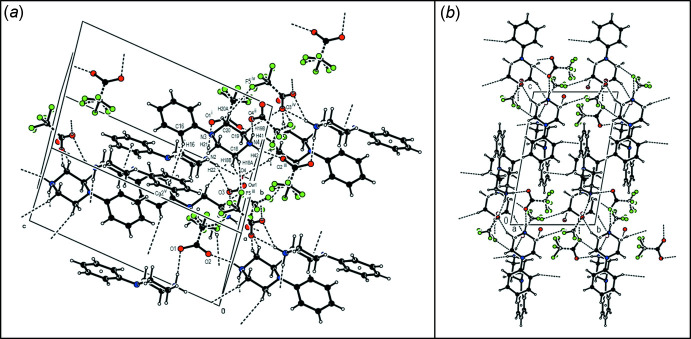
Parts of the crystal structure of compound (**IV**) showing (*a*) a general view of the C—H⋯O, N—H⋯O and C—H⋯F hydrogen bonds and C—H⋯π inter­actions and (*b*) the mol­ecular packing of (**IV**) down the *a*-axis. Hydrogen bonds and C—H⋯π inter­actions are drawn as dashed lines.

In the crystal structure of (**IV**), the cations and the anions are connected by C—H⋯O, N—H⋯O and C—H⋯F hydrogen bonds (Table 4 ▸, Fig. 8 ▸ *a*) and C—H⋯π inter­actions, generating sheets parallel to the (100) plane (Table 4 ▸, Fig. 8 ▸). These sheets further inter­act to maintain the crystal structure by linking through the oxygen atoms of water mol­ecules and by van der Waals inter­actions. As shown in Table 4 ▸, the main interactions in the structure of (**IV**) involve the oxygen atoms of carboxylate groups, while the 0.12 fraction of the water molecule contributes with one interaction of the type C—H⋯O and it is weak in comparison to the other oxygen-based ones.

## 4. Database survey

A search of the Cambridge Structural Database (Version 2020.3, last update February 2022; Groom *et al.*, 2016 ▸) for an unsubstituted 4-phenyl­piperazin-1-ium cation and *para-*substituted benzoate anion involved in the reported salts (**I**)–(**III**) gave no hits. However, searching for a branched phenyl piperazinium cation and *para-*substituted benzoate anion gave comparable hits, namely; 4-(4-meth­oxy­phen­yl)piperazin-1-ium 4-fluoro­benzoate monohydrate, 4-(4-meth­oxy­phen­yl)piperazin-1-ium 4-chloro­benzoate monohydrate, 4-(4-meth­oxy­phen­yl)piperazin-1-ium 4-bromo­benzoate monohydrate (FOVPOY, FOVPUE, FOVQAL; Kiran Kumar *et al.*, 2019*a* ▸), 4-(4-meth­oxy­phen­yl)piperazin-1-ium 4-iodo­benzoate monohydrate (KUJPUD; Kiran Kumar *et al.*, 2020*b* ▸). They exhibit a meth­oxy group as a substituent in the 4-phenyl­piperazin-1-ium cation while the reported compounds (**I**)–(**IV**) have no substituent. They also crystallize as monohydrates, and their crystal structures are based on differently sized chains of rings formed *via* a combination of hydrogen bonds of the type N–H⋯O and O–H⋯O and other weak inter­actions of types C—H⋯O and C—H⋯π to form sheets. In 4-(4-meth­oxy­phen­yl)piperazin-1-ium 4-amino­benzoate monohydrate (IHIMEU; Kiran Kumar *et al.*, 2020*a* ▸) the presence of the amino substituent in the anion, which acts as both a donor and as an acceptor of hydrogen bonds, makes the supra­molecular assembly of this compound more complex than those reported here. A search for 4-phenyl­piperazin-1-ium and acetate derivatives involved in the reported compound (**IV**) gave no hits.

## 5. Synthesis and crystallization

For the synthesis of salts (**I**)–(**IV**), a solution of commercially available (from Sigma–Aldrich) 1-phenyl­piperazine (100 mg, 0.62 mol) in methanol (10 ml) was mixed with equimolar solutions of the appropriate organic acids in methanol (10 ml) *viz.*, 4-eth­oxy­benzoic acid (103 mg, 0.62 mol) for (**I**), 4-meth­oxy­benzoic acid (94 mg, 0.62 mol) for (**II**), 4-methyl­benzoic acid (84 mg, 0.62 mol) for (**III**) and tri­fluoro­acetic acid (71 mg, 0.62 mol) for (**IV**). The corresponding solutions were stirred for 15 min at room temperature and allowed to stand at the same temperature. X-ray quality crystals were formed on slow evaporation in a week for all compounds, where ethanol:ethyl­acetate (1:1) was used for crystallization. The corresponding melting points were 353–355 K for (**I**), 368–370 K for (**II**), 338–340 K for (**III**) and 385–387 K for (**IV**).

## 6. Refinement

Crystal data, data collection and structure refinement details are summarized in Table 5 ▸. All H atoms bonded to C atoms were fixed geometrically and treated as riding with C—H = 0.93 Å (aromatic), C—H = 0.96 Å (meth­yl) or 0.97 Å (methyl­ene), with *U*_iso_(H) = 1.2*U*_eq_(C) or 1.5*U*_eq_(C). For the H atoms bonded to the N and O atoms, the atomic coordinates were refined with *U*_iso_(H) = 1.2*U*_eq_(N) and 1.5*U*_eq_(O), [for (**I**), N2—H*N*2 = 0.931 (19), N2—H*N*1 = 0.888 (17) Å and O*W*1—H*W*2 = 0.91 (3), O*W*1—H*W*1 = 0.88 (3) Å; for (**II**), N2—H*N*1 = 0.927 (16), N2—H*N*2 = 0.931 (18) Å and O*W*—H*W*1 = 0.840 (19), O*W*1—H*W*2 = 0.85 (2) Å; for (**III**), N2—H*N*1 = 0.900 (16), N2—H*N*2 = 0.918 (17) Å and O*W*1—H*W*1 = 0.89 (3), O*W*1—H*W*2 = 0.84 (2) Å and for (**IV**), N2—H22 = 0.87 (2) and N2—H21 = 0.88 (3) Å]. In (**IV**), the atoms of the CF_3_ groups of two tri­fluoro­acetate anions (B1, B2) are disordered over two sets of sites with site occupancies of 0.737 (3) and 0.263 (3). The corresponding bond distances in the disordered groups were restrained to be equal. The *U* ^ij^ components of these atoms were restrained to be equal and were restrained to approximate isotropic behaviour. The O*W*1 water molecule was refined with a resulting occupation factor of 0.245 (10) and the H atoms of the water molecule were placed geometrically.

## Supplementary Material

Crystal structure: contains datablock(s) global, I, II, III, IV. DOI: 10.1107/S2056989022006004/dj2048sup1.cif

Structure factors: contains datablock(s) I. DOI: 10.1107/S2056989022006004/dj2048Isup2.hkl

Supporting information file. DOI: 10.1107/S2056989022006004/dj2048Isup6.cml

Structure factors: contains datablock(s) II. DOI: 10.1107/S2056989022006004/dj2048IIsup3.hkl

Supporting information file. DOI: 10.1107/S2056989022006004/dj2048IIsup7.cml

Structure factors: contains datablock(s) III. DOI: 10.1107/S2056989022006004/dj2048IIIsup4.hkl

Supporting information file. DOI: 10.1107/S2056989022006004/dj2048IIIsup8.cml

Structure factors: contains datablock(s) IV. DOI: 10.1107/S2056989022006004/dj2048IVsup5.hkl

Supporting information file. DOI: 10.1107/S2056989022006004/dj2048IVsup9.cml

CCDC references: 2177037, 2177036, 2177035, 2177034

Additional supporting information:  crystallographic information; 3D view; checkCIF report

## Figures and Tables

**Table 1:** Hydrogen-bond geometry (Å, °) for (**I**) *Cg*1 and *Cg*3 are the centroids of the C12–C17 and C1–C6 benzene rings, respectively.

*D*—H⋯*A*	*D*—H	H⋯*A*	*D*⋯*A*	*D*—H⋯*A*
N2—H*N*1⋯O*W*1^i^	0.89 (2)	1.94 (2)	2.817 (3)	167 (2)
N2—H*N*2⋯O1	0.93 (2)	1.80 (2)	2.724 (3)	174 (2)
O*W*1—H*W*1⋯O2	0.88 (3)	1.75 (3)	2.630 (3)	178 (4)
O*W*1—H*W*2⋯O1^ii^	0.91 (3)	1.89 (3)	2.789 (3)	167 (3)
C9—H9*A*⋯O*W*1	0.97	2.52	3.308 (3)	138
C1—H1⋯*Cg*1^iii^	0.93	2.91	3.607 (3)	133
C5—H5⋯*Cg*1^iv^	0.93	2.79	3.570 (3)	142
C18—H18*B*⋯*Cg*3^v^	0.97	2.88	3.737 (4)	148

Symmetry codes: (i) 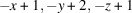 ; (ii) 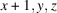 ; (iii) 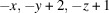 ; (iv) 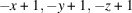 ; (v) 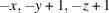 .

**Table 2:** Hydrogen-bond geometry (Å, °) for (**II**) *Cg*3 is the centroid of the C12–C17 benzene ring.

*D*—H⋯*A*	*D*—H	H⋯*A*	*D*⋯*A*	*D*—H⋯*A*
N2—H*N*1⋯O*W*1^i^	0.93 (2)	1.91 (2)	2.815 (2)	166 (2)
O*W*1—H*W*1⋯O1^ii^	0.84 (2)	1.80 (2)	2.633 (2)	175 (2)
N2—H*N*2⋯O2	0.93 (2)	1.81 (2)	2.7350 (19)	176 (2)
O*W*1—H*W*2⋯O2^iii^	0.85 (2)	1.96 (2)	2.7876 (19)	168 (2)
C8—H8*B*⋯O*W*1^ii^	0.97	2.53	3.331 (2)	140
C1—H1⋯*Cg*3^ii^	0.93	2.76	3.549 (2)	144
C5—H5⋯*Cg*3^iv^	0.93	2.86	3.625 (2)	140

Symmetry codes: (i) 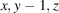 ; (ii) 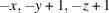 ; (iii) 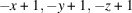 ; (iv) 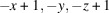 .

**Table 3:** Hydrogen-bond geometry (Å, °) for (**III**)

*D*—H⋯*A*	*D*—H	H⋯*A*	*D*⋯*A*	*D*—H⋯*A*
O*W*1—H*W*1⋯O2^i^	0.89 (3)	1.90 (3)	2.782 (2)	171 (4)
O*W*1—H*W*2⋯O1	0.84 (2)	1.92 (3)	2.751 (2)	172 (3)
N2—H*N*1⋯O1^ii^	0.90 (2)	1.94 (2)	2.819 (2)	164 (2)
N2—H*N*2⋯O2	0.92 (2)	1.80 (2)	2.7207 (19)	176 (2)
C8—H8*A*⋯O*W*1	0.97	2.33	3.116 (3)	138

Symmetry codes: (i) 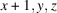 ; (ii) 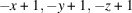 .

**Table 4:** Hydrogen-bond geometry (Å, °) for (**IV**) *Cg*2 is the centroid of the C1–C6 phenyl ring.

*D*—H⋯*A*	*D*—H	H⋯*A*	*D*⋯*A*	*D*—H⋯*A*
N2—H21⋯O1^i^	0.88	1.91	2.790 (4)	174
N2—H22⋯O3	0.87	2.04	2.860 (4)	157
N2—H22⋯O4	0.87	2.47	3.164 (5)	137
N4—H41⋯O4^ii^	0.86	1.95	2.759 (6)	156
N4—H42⋯O2^iii^	0.89	1.90	2.758 (4)	164
C18—H18*A*⋯F5′^iii^	0.97	2.53	3.273 (18)	134
C18—H18*B*⋯Ow1	0.97	2.08	2.929 (15)	145
C19—H19*B*⋯O3^iv^	0.97	2.59	3.420 (5)	144
C20—H20*A*⋯F5^iv^	0.97	2.64	3.468 (8)	144
C16—H16⋯*Cg*2^v^	0.93	2.99	3.745 (4)	140

Symmetry codes: (i) 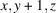 ; (ii) 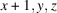 ; (iii) 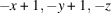 ; (iv) 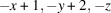 ; (v) 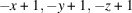 .

**Table 5:** Experimental details

	(**I**)	(**II**)	(**III**)	(**IV**)
Crystal data				
Chemical formula	C_10_H_15_N_2_^+^·C_9_H_9_O_3_^−^·H_2_O	C_10_H_15_N_2_^+^·C_8_H_7_O_3_^−^·H_2_O	C_10_H_15_N_2_^+^·C_8_H_7_O_2_^−^·H_2_O	C_10_H_15_N_2_^+^·C_2_F_3_O_2_^−^·0.123H_2_O
*M*_r_	346.42	332.39	316.39	278.47
Crystal system, space group	Triclinic, *P* 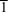	Triclinic, *P* 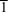	Triclinic, *P* 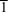	Triclinic, *P* 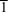
Temperature (K)	293	293	293	293
*a*, *b*, *c* (Å)	6.1635 (5), 7.5946 (6), 20.458 (2)	6.2039 (4), 7.5565 (7), 18.614 (1)	6.1175 (5), 7.6225 (7), 18.452 (1)	9.6544 (6), 9.9029 (6), 15.2090 (9)
α, β, γ (°)	79.545 (7), 86.521 (7), 83.791 (7)	81.799 (7), 87.020 (7), 84.852 (7)	97.421 (9), 90.403 (8), 92.405 (8)	79.621 (6), 86.579 (6), 70.603 (6)
*V* (Å^3^)	935.38 (14)	859.53 (11)	852.40 (12)	1349.10 (15)
*Z*	2	2	2	4
Radiation type	Mo *K*α	Mo *K*α	Mo *K*α	Mo *K*α
μ (mm^−1^)	0.09	0.09	0.08	0.12
Crystal size (mm)	0.48 × 0.42 × 0.1	0.48 × 0.48 × 0.32	0.5 × 0.4 × 0.08	0.48 × 0.48 × 0.36

Data collection				
Diffractometer	Oxford Diffraction Xcalibur	Oxford Diffraction Xcalibur	Oxford Diffraction Xcalibur	Oxford Diffraction Xcalibur
Absorption correction	Multi-scan (*CrysAlis RED*; Oxford Diffraction, 2009 ▸)	Multi-scan (*CrysAlis RED*; Oxford Diffraction, 2009 ▸)	Multi-scan (*CrysAlis RED*; Oxford Diffraction, 2009 ▸)	Multi-scan (*CrysAlis RED*; Oxford Diffraction, 2009 ▸)
*T*_min_, *T*_max_	0.623, 1.000	0.520, 1.000	0.837, 1.000	0.724, 1.000
No. of measured, independent and observed [*I* > 2σ(*I*)] reflections	5989, 3429, 2159	5360, 3142, 2322	5354, 3126, 2248	9220, 4940, 2777
*R*_int_	0.022	0.016	0.013	0.014
(sin θ/λ)_max_ (Å^−1^)	0.602	0.602	0.602	0.602

Refinement				
*R*[*F* ^2^ > 2σ(*F* ^2^)], *wR*(*F* ^2^), *S*	0.054, 0.124, 1.08	0.045, 0.125, 1.06	0.046, 0.128, 1.03	0.070, 0.235, 1.07
No. of reflections	3424	3139	3118	4927
No. of parameters	244	230	226	375
No. of restraints	2	4	4	4
H-atom treatment	H atoms treated by a mixture of independent and constrained refinement	H atoms treated by a mixture of independent and constrained refinement	H atoms treated by a mixture of independent and constrained refinement	H-atom parameters constrained
Δρ_max_, Δρ_min_ (e Å^−3^)	0.15, −0.15	0.2, −0.17	0.16, −0.16	0.42, −0.28

Computer programs: *CrysAlis CCD* (Oxford Diffraction, 2009 ▸), *CrysAlis RED* (Oxford Diffraction, 2009 ▸), *SHELXT* (Sheldrick, 2015*a* ▸), *SHELXL2014* (Sheldrick, 2015*b* ▸), *Mercury* (Macrae *et al.*, 2020 ▸), *PLATON* (Spek, 2020 ▸) and *publCIF* (Westrip, 2010 ▸).

## supplementary crystallographic information

### 4-Phenylpiperazin-1-ium 4-ethoxybenzoate monohydrate (I) Crystal data

**Table:**

C_10_H_15_N_2_^+^·C_9_H_9_O_3_^−^·H_2_O	*Z* = 2
*M**_r_* = 346.42	*F*(000) = 372
Triclinic, *P*1	*D*_x_ = 1.23 Mg m^−^^3^
Hall symbol: -P 1	Mo *K*α radiation, λ = 0.71073 Å
*a* = 6.1635 (5) Å	Cell parameters from 2352 reflections
*b* = 7.5946 (6) Å	θ = 3.0–27.8°
*c* = 20.458 (2) Å	µ = 0.09 mm^−^^1^
α = 79.545 (7)°	*T* = 293 K
β = 86.521 (7)°	Plate, colourless
γ = 83.791 (7)°	0.48 × 0.42 × 0.1 mm
*V* = 935.38 (14) Å^3^	

### 4-Phenylpiperazin-1-ium 4-ethoxybenzoate monohydrate (I) Data collection

**Table:**

Oxford Diffraction Xcalibur diffractometer	2159 reflections with *I* > 2σ(*I*)
ω scans	*R*_int_ = 0.022
Absorption correction: multi-scan (CrysAlis RED; Oxford Diffraction, 2009)	θ_max_ = 25.4°, θ_min_ = 3.0°
*T*_min_ = 0.623, *T*_max_ = 1.000	*h* = −7→7
5989 measured reflections	*k* = −9→9
3429 independent reflections	*l* = −24→14

### 4-Phenylpiperazin-1-ium 4-ethoxybenzoate monohydrate (I) Refinement

**Table:**

Refinement on *F*^2^	Secondary atom site location: structure-invariant direct methods
Least-squares matrix: full	Hydrogen site location: mixed
*R*[*F*^2^ > 2σ(*F*^2^)] = 0.054	H atoms treated by a mixture of independent and constrained refinement
*wR*(*F*^2^) = 0.124	*w* = 1/[σ^2^(*F*_o_^2^) + (0.0392*P*)^2^ + 0.2859*P*] where *P* = (*F*_o_^2^ + 2*F*_c_^2^)/3
*S* = 1.08	(Δ/σ)_max_ < 0.001
3424 reflections	Δρ_max_ = 0.15 e Å^−^^3^
244 parameters	Δρ_min_ = −0.15 e Å^−^^3^
2 restraints	Extinction correction: SHELXL2018/3 (Sheldrick 2015b), Fc^*^=kFc[1+0.001xFc^2^λ^3^/sin(2θ)]^-1/4^
0 constraints	Extinction coefficient: 0.0058 (17)
Primary atom site location: structure-invariant direct methods	

### 4-Phenylpiperazin-1-ium 4-ethoxybenzoate monohydrate (I) Special details

**Table:**

Geometry. All esds (except the esd in the dihedral angle between two l.s. planes) are estimated using the full covariance matrix. The cell esds are taken into account individually in the estimation of esds in distances, angles and torsion angles; correlations between esds in cell parameters are only used when they are defined by crystal symmetry. An approximate (isotropic) treatment of cell esds is used for estimating esds involving l.s. planes.

### 4-Phenylpiperazin-1-ium 4-ethoxybenzoate monohydrate (I) Fractional atomic coordinates and isotropic or equivalent isotropic displacement parameters (Å^2^)

**Table:**

	*x*	*y*	*z*	*U*_iso_*/*U*_eq_	
C1	0.1990 (4)	0.7508 (4)	0.79338 (12)	0.0626 (7)	
H1	0.069252	0.809989	0.776125	0.075*	
C2	0.2437 (5)	0.7539 (4)	0.85869 (13)	0.0782 (9)	
H2	0.142853	0.814295	0.884641	0.094*	
C3	0.4331 (5)	0.6699 (4)	0.88559 (14)	0.0759 (9)	
H3	0.462163	0.672073	0.929504	0.091*	
C4	0.5786 (5)	0.5827 (4)	0.84658 (14)	0.0753 (8)	
H4	0.709095	0.525999	0.864083	0.09*	
C5	0.5363 (4)	0.5771 (3)	0.78183 (12)	0.0579 (7)	
H5	0.638106	0.515837	0.756445	0.069*	
C6	0.3432 (3)	0.6615 (3)	0.75355 (10)	0.0407 (5)	
C7	0.1046 (3)	0.7635 (3)	0.66062 (11)	0.0484 (6)	
H7A	0.130663	0.888733	0.656771	0.058*	
H7B	−0.01969	0.742138	0.691398	0.058*	
C8	0.0502 (3)	0.7292 (3)	0.59343 (11)	0.0496 (6)	
H8A	0.005085	0.609001	0.59798	0.059*	
H8B	−0.07032	0.814458	0.576145	0.059*	
C9	0.4290 (3)	0.6216 (3)	0.57366 (10)	0.0480 (6)	
H9A	0.555062	0.634835	0.543053	0.058*	
H9B	0.392361	0.498713	0.578625	0.058*	
C10	0.4839 (3)	0.6594 (3)	0.64012 (10)	0.0447 (6)	
H10A	0.602615	0.572985	0.657958	0.054*	
H10B	0.5333	0.778458	0.634186	0.054*	
C11	0.2356 (4)	0.7988 (3)	0.37750 (13)	0.0504 (6)	
C12	0.1740 (3)	0.8093 (3)	0.30715 (11)	0.0409 (5)	
C13	−0.0108 (3)	0.7381 (3)	0.29259 (11)	0.0462 (6)	
H13	−0.09987	0.685018	0.327326	0.055*	
C14	−0.0669 (4)	0.7436 (3)	0.22762 (11)	0.0496 (6)	
H14	−0.191724	0.694349	0.218988	0.06*	
C15	0.0633 (4)	0.8223 (3)	0.17610 (11)	0.0480 (6)	
C16	0.2462 (4)	0.8988 (3)	0.18948 (12)	0.0547 (6)	
H16	0.332264	0.955074	0.154649	0.066*	
C17	0.3005 (4)	0.8916 (3)	0.25403 (12)	0.0521 (6)	
H17	0.424178	0.942678	0.262421	0.063*	
C18	−0.1614 (5)	0.7562 (5)	0.09410 (14)	0.0931 (10)	
H18A	−0.293805	0.818359	0.110377	0.112*	
H18B	−0.155291	0.63023	0.114607	0.112*	
C19	−0.1605 (8)	0.7747 (7)	0.02032 (17)	0.178 (2)	
H19A	−0.195823	0.898767	0.00101	0.268*	
H19B	−0.266871	0.703796	0.008494	0.268*	
H19C	−0.018288	0.73364	0.003915	0.268*	
N1	0.2969 (3)	0.6493 (2)	0.68767 (8)	0.0382 (4)	
N2	0.2420 (3)	0.7474 (3)	0.54621 (10)	0.0452 (5)	
O1	0.1211 (3)	0.7133 (2)	0.42359 (8)	0.0622 (5)	
O2	0.3970 (3)	0.8727 (3)	0.38724 (10)	0.0885 (7)	
O3	0.0253 (3)	0.8330 (2)	0.11014 (8)	0.0681 (5)	
OW1	0.7230 (4)	0.8819 (2)	0.46338 (9)	0.0591 (5)	
HN1	0.275 (4)	0.860 (2)	0.5421 (11)	0.056 (7)*	
HN2	0.208 (4)	0.729 (3)	0.5044 (9)	0.067 (8)*	
HW1	0.612 (5)	0.879 (4)	0.4385 (15)	0.102 (11)*	
HW2	0.842 (5)	0.825 (4)	0.4445 (15)	0.102 (12)*	

### 4-Phenylpiperazin-1-ium 4-ethoxybenzoate monohydrate (I) Atomic displacement parameters (Å^2^)

**Table:**

	*U*^11^	*U*^22^	*U*^33^	*U*^12^	*U*^13^	*U*^23^
C1	0.0719 (17)	0.0673 (18)	0.0465 (15)	0.0151 (14)	−0.0066 (13)	−0.0156 (13)
C2	0.103 (2)	0.079 (2)	0.0522 (17)	0.0150 (18)	−0.0020 (16)	−0.0248 (15)
C3	0.106 (2)	0.076 (2)	0.0510 (17)	−0.0055 (18)	−0.0223 (17)	−0.0207 (15)
C4	0.0735 (18)	0.090 (2)	0.0636 (18)	0.0027 (16)	−0.0267 (15)	−0.0145 (17)
C5	0.0561 (15)	0.0696 (18)	0.0486 (15)	0.0031 (13)	−0.0116 (12)	−0.0147 (13)
C6	0.0467 (12)	0.0353 (13)	0.0414 (13)	−0.0092 (10)	−0.0045 (10)	−0.0067 (10)
C7	0.0399 (12)	0.0595 (16)	0.0441 (13)	0.0007 (11)	−0.0025 (10)	−0.0078 (11)
C8	0.0407 (12)	0.0622 (16)	0.0445 (14)	−0.0045 (11)	−0.0053 (10)	−0.0053 (11)
C9	0.0480 (13)	0.0512 (15)	0.0423 (13)	−0.0011 (11)	0.0026 (10)	−0.0057 (11)
C10	0.0396 (12)	0.0496 (14)	0.0435 (13)	−0.0024 (10)	−0.0018 (10)	−0.0062 (11)
C11	0.0524 (14)	0.0427 (14)	0.0593 (17)	0.0050 (12)	−0.0162 (13)	−0.0190 (12)
C12	0.0398 (12)	0.0373 (13)	0.0475 (13)	−0.0007 (10)	−0.0072 (10)	−0.0121 (10)
C13	0.0494 (13)	0.0462 (14)	0.0432 (13)	−0.0111 (11)	−0.0024 (11)	−0.0048 (11)
C14	0.0502 (13)	0.0519 (15)	0.0490 (14)	−0.0163 (11)	−0.0091 (11)	−0.0063 (12)
C15	0.0556 (14)	0.0469 (15)	0.0412 (14)	−0.0042 (11)	−0.0052 (11)	−0.0065 (11)
C16	0.0534 (14)	0.0566 (16)	0.0533 (16)	−0.0136 (12)	0.0088 (12)	−0.0063 (12)
C17	0.0409 (12)	0.0533 (16)	0.0659 (17)	−0.0098 (11)	−0.0026 (12)	−0.0174 (13)
C18	0.111 (2)	0.121 (3)	0.0572 (18)	−0.046 (2)	−0.0218 (17)	−0.0148 (18)
C19	0.242 (6)	0.251 (6)	0.063 (2)	−0.137 (5)	−0.039 (3)	−0.007 (3)
N1	0.0374 (9)	0.0419 (11)	0.0350 (10)	−0.0028 (8)	−0.0021 (8)	−0.0062 (8)
N2	0.0548 (12)	0.0428 (13)	0.0390 (11)	−0.0088 (10)	−0.0079 (9)	−0.0058 (9)
O1	0.0706 (11)	0.0703 (12)	0.0465 (10)	−0.0048 (10)	−0.0127 (9)	−0.0108 (9)
O2	0.0835 (13)	0.1109 (17)	0.0825 (14)	−0.0368 (12)	−0.0321 (11)	−0.0215 (12)
O3	0.0816 (12)	0.0792 (13)	0.0450 (10)	−0.0182 (10)	−0.0052 (9)	−0.0080 (9)
OW1	0.0593 (11)	0.0598 (12)	0.0604 (12)	−0.0122 (10)	−0.0135 (10)	−0.0092 (9)

### 4-Phenylpiperazin-1-ium 4-ethoxybenzoate monohydrate (I) Geometric parameters (Å, º)

**Table:**

C1—C6	1.377 (3)	C11—O2	1.237 (3)
C1—C2	1.385 (3)	C11—O1	1.267 (3)
C1—H1	0.93	C11—C12	1.497 (3)
C2—C3	1.365 (4)	C12—C13	1.383 (3)
C2—H2	0.93	C12—C17	1.391 (3)
C3—C4	1.362 (4)	C13—C14	1.386 (3)
C3—H3	0.93	C13—H13	0.93
C4—C5	1.375 (3)	C14—C15	1.373 (3)
C4—H4	0.93	C14—H14	0.93
C5—C6	1.395 (3)	C15—O3	1.370 (3)
C5—H5	0.93	C15—C16	1.386 (3)
C6—N1	1.416 (3)	C16—C17	1.372 (3)
C7—N1	1.465 (2)	C16—H16	0.93
C7—C8	1.508 (3)	C17—H17	0.93
C7—H7A	0.97	C18—O3	1.425 (3)
C7—H7B	0.97	C18—C19	1.490 (4)
C8—N2	1.482 (3)	C18—H18A	0.97
C8—H8A	0.97	C18—H18B	0.97
C8—H8B	0.97	C19—H19A	0.96
C9—N2	1.484 (3)	C19—H19B	0.96
C9—C10	1.504 (3)	C19—H19C	0.96
C9—H9A	0.97	N2—HN1	0.887 (16)
C9—H9B	0.97	N2—HN2	0.930 (16)
C10—N1	1.462 (3)	OW1—HW1	0.88 (3)
C10—H10A	0.97	OW1—HW2	0.91 (3)
C10—H10B	0.97		
			
C6—C1—C2	121.2 (2)	O2—C11—C12	118.0 (2)
C6—C1—H1	119.4	O1—C11—C12	118.2 (2)
C2—C1—H1	119.4	C13—C12—C17	117.6 (2)
C3—C2—C1	121.1 (3)	C13—C12—C11	121.3 (2)
C3—C2—H2	119.4	C17—C12—C11	121.1 (2)
C1—C2—H2	119.4	C12—C13—C14	121.8 (2)
C4—C3—C2	118.4 (3)	C12—C13—H13	119.1
C4—C3—H3	120.8	C14—C13—H13	119.1
C2—C3—H3	120.8	C15—C14—C13	119.4 (2)
C3—C4—C5	121.3 (3)	C15—C14—H14	120.3
C3—C4—H4	119.4	C13—C14—H14	120.3
C5—C4—H4	119.4	O3—C15—C14	124.4 (2)
C4—C5—C6	121.1 (2)	O3—C15—C16	115.8 (2)
C4—C5—H5	119.4	C14—C15—C16	119.8 (2)
C6—C5—H5	119.4	C17—C16—C15	120.1 (2)
C1—C6—C5	116.9 (2)	C17—C16—H16	119.9
C1—C6—N1	122.23 (19)	C15—C16—H16	119.9
C5—C6—N1	120.8 (2)	C16—C17—C12	121.2 (2)
N1—C7—C8	112.74 (18)	C16—C17—H17	119.4
N1—C7—H7A	109	C12—C17—H17	119.4
C8—C7—H7A	109	O3—C18—C19	107.7 (3)
N1—C7—H7B	109	O3—C18—H18A	110.2
C8—C7—H7B	109	C19—C18—H18A	110.2
H7A—C7—H7B	107.8	O3—C18—H18B	110.2
N2—C8—C7	110.70 (17)	C19—C18—H18B	110.2
N2—C8—H8A	109.5	H18A—C18—H18B	108.5
C7—C8—H8A	109.5	C18—C19—H19A	109.5
N2—C8—H8B	109.5	C18—C19—H19B	109.5
C7—C8—H8B	109.5	H19A—C19—H19B	109.5
H8A—C8—H8B	108.1	C18—C19—H19C	109.5
N2—C9—C10	110.47 (18)	H19A—C19—H19C	109.5
N2—C9—H9A	109.6	H19B—C19—H19C	109.5
C10—C9—H9A	109.6	C6—N1—C10	115.21 (16)
N2—C9—H9B	109.6	C6—N1—C7	115.50 (17)
C10—C9—H9B	109.6	C10—N1—C7	111.72 (16)
H9A—C9—H9B	108.1	C8—N2—C9	109.60 (17)
N1—C10—C9	112.16 (17)	C8—N2—HN1	106.9 (15)
N1—C10—H10A	109.2	C9—N2—HN1	109.4 (15)
C9—C10—H10A	109.2	C8—N2—HN2	110.8 (15)
N1—C10—H10B	109.2	C9—N2—HN2	112.2 (15)
C9—C10—H10B	109.2	HN1—N2—HN2	108 (2)
H10A—C10—H10B	107.9	C15—O3—C18	117.7 (2)
O2—C11—O1	123.7 (2)	HW1—OW1—HW2	107 (3)
			
C6—C1—C2—C3	0.6 (4)	O3—C15—C16—C17	−178.7 (2)
C1—C2—C3—C4	0.2 (5)	C14—C15—C16—C17	1.8 (4)
C2—C3—C4—C5	−0.7 (5)	C15—C16—C17—C12	−0.4 (4)
C3—C4—C5—C6	0.5 (4)	C13—C12—C17—C16	−1.2 (3)
C2—C1—C6—C5	−0.8 (4)	C11—C12—C17—C16	178.9 (2)
C2—C1—C6—N1	177.0 (2)	C1—C6—N1—C10	143.0 (2)
C4—C5—C6—C1	0.3 (4)	C5—C6—N1—C10	−39.3 (3)
C4—C5—C6—N1	−177.6 (2)	C1—C6—N1—C7	10.3 (3)
N1—C7—C8—N2	−54.6 (3)	C5—C6—N1—C7	−171.9 (2)
N2—C9—C10—N1	56.7 (2)	C9—C10—N1—C6	172.59 (17)
O2—C11—C12—C13	−176.6 (2)	C9—C10—N1—C7	−53.0 (2)
O1—C11—C12—C13	4.1 (3)	C8—C7—N1—C6	−173.71 (18)
O2—C11—C12—C17	3.3 (3)	C8—C7—N1—C10	52.0 (2)
O1—C11—C12—C17	−176.0 (2)	C7—C8—N2—C9	57.2 (2)
C17—C12—C13—C14	1.6 (3)	C10—C9—N2—C8	−58.4 (2)
C11—C12—C13—C14	−178.6 (2)	C14—C15—O3—C18	−0.4 (4)
C12—C13—C14—C15	−0.3 (3)	C16—C15—O3—C18	−179.9 (2)
C13—C14—C15—O3	179.1 (2)	C19—C18—O3—C15	−176.7 (3)
C13—C14—C15—C16	−1.4 (3)		

### 4-Phenylpiperazin-1-ium 4-ethoxybenzoate monohydrate (I) Hydrogen-bond geometry (Å, º)

*Cg*1 and *Cg*3 are the centroids of the C12–C17 and C1–C6 benzene rings, respectively.

**Table:**

*D*—H···*A*	*D*—H	H···*A*	*D*···*A*	*D*—H···*A*
N2—H*N*1···O*W*1^i^	0.89 (2)	1.94 (2)	2.817 (3)	167 (2)
N2—H*N*2···O1	0.93 (2)	1.80 (2)	2.724 (3)	174 (2)
O*W*1—H*W*1···O2	0.88 (3)	1.75 (3)	2.630 (3)	178 (4)
O*W*1—H*W*2···O1^ii^	0.91 (3)	1.89 (3)	2.789 (3)	167 (3)
C9—H9*A*···O*W*1	0.97	2.52	3.308 (3)	138
C1—H1···*Cg*1^iii^	0.93	2.91	3.607 (3)	133
C5—H5···*Cg*1^iv^	0.93	2.79	3.570 (3)	142
C18—H18*B*···*Cg*3^v^	0.97	2.88	3.737 (4)	148

Symmetry codes: (i) −*x*+1, −*y*+2, −*z*+1; (ii) *x*+1, *y*, *z*; (iii) −*x*, −*y*+2, −*z*+1; (iv) −*x*+1, −*y*+1, −*z*+1; (v) −*x*, −*y*+1, −*z*+1.

### 4-Phenylpiperazin-1-ium 4-methoxybenzoate monohydrate (II) Crystal data

**Table:**

C_10_H_15_N_2_^+^·C_8_H_7_O_3_^−^·H_2_O	*Z* = 2
*M**_r_* = 332.39	*F*(000) = 356
Triclinic, *P*1	*D*_x_ = 1.284 Mg m^−^^3^
Hall symbol: -P 1	Mo *K*α radiation, λ = 0.71073 Å
*a* = 6.2039 (4) Å	Cell parameters from 2855 reflections
*b* = 7.5565 (7) Å	θ = 3.1–27.8°
*c* = 18.614 (1) Å	µ = 0.09 mm^−^^1^
α = 81.799 (7)°	*T* = 293 K
β = 87.020 (7)°	Prism, colourless
γ = 84.852 (7)°	0.48 × 0.48 × 0.32 mm
*V* = 859.53 (11) Å^3^	

### 4-Phenylpiperazin-1-ium 4-methoxybenzoate monohydrate (II) Data collection

**Table:**

Oxford Diffraction Xcalibur diffractometer	2322 reflections with *I* > 2σ(*I*)
ω scans	*R*_int_ = 0.016
Absorption correction: multi-scan (CrysAlis RED; Oxford Diffraction, 2009)	θ_max_ = 25.3°, θ_min_ = 3.1°
*T*_min_ = 0.520, *T*_max_ = 1.000	*h* = −7→5
5360 measured reflections	*k* = −9→9
3142 independent reflections	*l* = −22→22

### 4-Phenylpiperazin-1-ium 4-methoxybenzoate monohydrate (II) Refinement

**Table:**

Refinement on *F*^2^	Secondary atom site location: structure-invariant direct methods
Least-squares matrix: full	Hydrogen site location: mixed
*R*[*F*^2^ > 2σ(*F*^2^)] = 0.045	H atoms treated by a mixture of independent and constrained refinement
*wR*(*F*^2^) = 0.125	*w* = 1/[σ^2^(*F*_o_^2^) + (0.0613*P*)^2^ + 0.1503*P*] where *P* = (*F*_o_^2^ + 2*F*_c_^2^)/3
*S* = 1.06	(Δ/σ)_max_ < 0.001
3139 reflections	Δρ_max_ = 0.2 e Å^−^^3^
230 parameters	Δρ_min_ = −0.16 e Å^−^^3^
4 restraints	Extinction correction: SHELXL2018/3 (Sheldrick 2015b), Fc^*^=kFc[1+0.001xFc^2^λ^3^/sin(2θ)]^-1/4^
0 constraints	Extinction coefficient: 0.032 (4)
Primary atom site location: structure-invariant direct methods	

### 4-Phenylpiperazin-1-ium 4-methoxybenzoate monohydrate (II) Special details

**Table:**

Geometry. All esds (except the esd in the dihedral angle between two l.s. planes) are estimated using the full covariance matrix. The cell esds are taken into account individually in the estimation of esds in distances, angles and torsion angles; correlations between esds in cell parameters are only used when they are defined by crystal symmetry. An approximate (isotropic) treatment of cell esds is used for estimating esds involving l.s. planes.

### 4-Phenylpiperazin-1-ium 4-methoxybenzoate monohydrate (II) Fractional atomic coordinates and isotropic or equivalent isotropic displacement parameters (Å^2^)

**Table:**

	*x*	*y*	*z*	*U*_iso_*/*U*_eq_	
C1	−0.0254 (3)	0.4086 (3)	0.19209 (10)	0.0516 (5)	
H1	−0.120858	0.476382	0.219629	0.062*	
C2	−0.0695 (3)	0.3996 (3)	0.12102 (11)	0.0625 (6)	
H2	−0.194898	0.46027	0.101533	0.075*	
C3	0.0686 (3)	0.3024 (3)	0.07854 (10)	0.0597 (6)	
H3	0.039957	0.298402	0.030279	0.072*	
C4	0.2497 (3)	0.2117 (3)	0.10901 (10)	0.0593 (6)	
H4	0.343702	0.143909	0.08102	0.071*	
C5	0.2964 (3)	0.2181 (3)	0.18024 (9)	0.0512 (5)	
H5	0.420732	0.154912	0.199433	0.061*	
C6	0.1594 (2)	0.3180 (2)	0.22353 (8)	0.0361 (4)	
C7	0.0222 (3)	0.3258 (2)	0.34797 (8)	0.0419 (4)	
H7A	−0.026461	0.205915	0.354319	0.05*	
H7B	−0.096084	0.40906	0.32889	0.05*	
C8	0.0795 (3)	0.3708 (2)	0.42029 (9)	0.0456 (4)	
H8A	0.116889	0.494094	0.414858	0.055*	
H8B	−0.044531	0.359357	0.454053	0.055*	
C9	0.4547 (3)	0.2652 (3)	0.39777 (9)	0.0485 (5)	
H9A	0.574598	0.182967	0.416336	0.058*	
H9B	0.499661	0.386146	0.392734	0.058*	
C10	0.3979 (3)	0.2229 (3)	0.32457 (9)	0.0445 (4)	
H10A	0.520581	0.241283	0.29068	0.053*	
H10B	0.36928	0.097655	0.328948	0.053*	
C11	0.2655 (3)	0.2146 (2)	0.63263 (10)	0.0474 (5)	
C12	0.3208 (3)	0.2073 (2)	0.71052 (9)	0.0389 (4)	
C13	0.1897 (3)	0.1251 (2)	0.76647 (10)	0.0479 (5)	
H13	0.067829	0.072733	0.755273	0.057*	
C14	0.2386 (3)	0.1207 (3)	0.83782 (10)	0.0496 (5)	
H14	0.149875	0.065178	0.874461	0.06*	
C15	0.4186 (3)	0.1982 (2)	0.85567 (9)	0.0442 (4)	
C16	0.5528 (3)	0.2784 (2)	0.80102 (9)	0.0453 (4)	
H16	0.675183	0.329713	0.812349	0.054*	
C17	0.5021 (3)	0.2808 (2)	0.72949 (9)	0.0424 (4)	
H17	0.592994	0.333766	0.6929	0.051*	
C18	0.6395 (4)	0.2628 (4)	0.94845 (12)	0.0858 (8)	
H13A	0.643815	0.248303	1.000449	0.129*	
H13B	0.76663	0.201465	0.928951	0.129*	
H13C	0.63455	0.388125	0.929616	0.129*	
N1	0.2079 (2)	0.33487 (18)	0.29565 (7)	0.0359 (3)	
N2	0.2652 (2)	0.2489 (2)	0.44963 (8)	0.0434 (4)	
O1	0.1058 (3)	0.1395 (3)	0.61995 (9)	0.0873 (6)	
O2	0.3847 (2)	0.29717 (19)	0.58441 (7)	0.0598 (4)	
O3	0.4517 (2)	0.1895 (2)	0.92816 (7)	0.0620 (4)	
OW1	0.2271 (2)	0.8777 (2)	0.46022 (8)	0.0579 (4)	
HN1	0.230 (3)	0.131 (2)	0.4565 (11)	0.07*	
HW1	0.120 (3)	0.865 (3)	0.4356 (11)	0.07*	
HN2	0.303 (3)	0.270 (3)	0.4956 (9)	0.07*	
HW2	0.336 (3)	0.822 (3)	0.4413 (12)	0.07*	

### 4-Phenylpiperazin-1-ium 4-methoxybenzoate monohydrate (II) Atomic displacement parameters (Å^2^)

**Table:**

	*U*^11^	*U*^22^	*U*^33^	*U*^12^	*U*^13^	*U*^23^
C1	0.0534 (11)	0.0579 (12)	0.0441 (10)	0.0127 (9)	−0.0106 (8)	−0.0169 (9)
C2	0.0654 (13)	0.0739 (14)	0.0491 (11)	0.0143 (11)	−0.0228 (10)	−0.0166 (10)
C3	0.0820 (14)	0.0654 (13)	0.0347 (10)	−0.0036 (11)	−0.0147 (10)	−0.0145 (9)
C4	0.0717 (13)	0.0660 (13)	0.0411 (10)	0.0070 (11)	0.0022 (9)	−0.0202 (9)
C5	0.0535 (11)	0.0597 (12)	0.0399 (10)	0.0105 (9)	−0.0032 (8)	−0.0137 (8)
C6	0.0405 (9)	0.0344 (9)	0.0346 (8)	−0.0042 (7)	−0.0035 (7)	−0.0071 (7)
C7	0.0388 (9)	0.0515 (11)	0.0352 (9)	0.0009 (8)	−0.0006 (7)	−0.0083 (7)
C8	0.0518 (10)	0.0495 (11)	0.0354 (9)	0.0016 (8)	0.0004 (8)	−0.0104 (8)
C9	0.0425 (10)	0.0619 (12)	0.0419 (10)	−0.0015 (8)	−0.0105 (8)	−0.0090 (8)
C10	0.0385 (9)	0.0569 (11)	0.0381 (9)	0.0049 (8)	−0.0053 (7)	−0.0113 (8)
C11	0.0484 (11)	0.0464 (11)	0.0508 (11)	0.0051 (8)	−0.0161 (9)	−0.0190 (9)
C12	0.0387 (9)	0.0356 (9)	0.0443 (9)	0.0023 (7)	−0.0086 (7)	−0.0131 (7)
C13	0.0366 (9)	0.0485 (11)	0.0617 (12)	−0.0058 (8)	−0.0057 (8)	−0.0157 (9)
C14	0.0437 (10)	0.0551 (12)	0.0500 (11)	−0.0060 (8)	0.0047 (8)	−0.0079 (9)
C15	0.0450 (10)	0.0485 (10)	0.0398 (9)	0.0020 (8)	−0.0042 (8)	−0.0110 (8)
C16	0.0461 (10)	0.0501 (11)	0.0430 (10)	−0.0103 (8)	−0.0101 (8)	−0.0112 (8)
C17	0.0447 (10)	0.0437 (10)	0.0400 (9)	−0.0072 (8)	−0.0036 (7)	−0.0075 (7)
C18	0.0785 (16)	0.136 (2)	0.0509 (13)	−0.0228 (16)	−0.0171 (11)	−0.0274 (14)
N1	0.0347 (7)	0.0416 (8)	0.0320 (7)	0.0006 (6)	−0.0034 (5)	−0.0091 (6)
N2	0.0553 (9)	0.0450 (8)	0.0319 (7)	−0.0061 (7)	−0.0089 (6)	−0.0082 (6)
O1	0.0815 (11)	0.1184 (15)	0.0727 (11)	−0.0352 (10)	−0.0327 (9)	−0.0230 (10)
O2	0.0721 (9)	0.0700 (10)	0.0402 (7)	−0.0038 (7)	−0.0139 (7)	−0.0144 (7)
O3	0.0641 (9)	0.0866 (11)	0.0365 (7)	−0.0067 (8)	−0.0047 (6)	−0.0117 (7)
OW1	0.0595 (9)	0.0631 (9)	0.0544 (8)	−0.0097 (7)	−0.0177 (7)	−0.0108 (7)

### 4-Phenylpiperazin-1-ium 4-methoxybenzoate monohydrate (II) Geometric parameters (Å, º)

**Table:**

C1—C2	1.377 (3)	C10—H10A	0.97
C1—C6	1.393 (2)	C10—H10B	0.97
C1—H1	0.93	C11—O1	1.234 (2)
C2—C3	1.371 (3)	C11—O2	1.263 (2)
C2—H2	0.93	C11—C12	1.499 (2)
C3—C4	1.368 (3)	C12—C17	1.381 (2)
C3—H3	0.93	C12—C13	1.396 (3)
C4—C5	1.380 (3)	C13—C14	1.373 (3)
C4—H4	0.93	C13—H13	0.93
C5—C6	1.388 (2)	C14—C15	1.383 (3)
C5—H5	0.93	C14—H14	0.93
C6—N1	1.4165 (19)	C15—O3	1.367 (2)
C7—N1	1.469 (2)	C15—C16	1.386 (2)
C7—C8	1.502 (2)	C16—C17	1.381 (2)
C7—H7A	0.97	C16—H16	0.93
C7—H7B	0.97	C17—H17	0.93
C8—N2	1.485 (2)	C18—O3	1.424 (3)
C8—H8A	0.97	C18—H13A	0.96
C8—H8B	0.97	C18—H13B	0.96
C9—N2	1.484 (2)	C18—H13C	0.96
C9—C10	1.509 (2)	N2—HN1	0.924 (15)
C9—H9A	0.97	N2—HN2	0.938 (16)
C9—H9B	0.97	OW1—HW1	0.847 (16)
C10—N1	1.467 (2)	OW1—HW2	0.850 (16)
			
C2—C1—C6	121.26 (16)	C9—C10—H10B	109.1
C2—C1—H1	119.4	H10A—C10—H10B	107.9
C6—C1—H1	119.4	O1—C11—O2	124.33 (18)
C3—C2—C1	121.01 (18)	O1—C11—C12	117.62 (19)
C3—C2—H2	119.5	O2—C11—C12	118.06 (16)
C1—C2—H2	119.5	C17—C12—C13	117.75 (16)
C4—C3—C2	118.25 (17)	C17—C12—C11	121.49 (16)
C4—C3—H3	120.9	C13—C12—C11	120.77 (16)
C2—C3—H3	120.9	C14—C13—C12	120.75 (16)
C3—C4—C5	121.67 (17)	C14—C13—H13	119.6
C3—C4—H4	119.2	C12—C13—H13	119.6
C5—C4—H4	119.2	C13—C14—C15	120.56 (17)
C4—C5—C6	120.65 (17)	C13—C14—H14	119.7
C4—C5—H5	119.7	C15—C14—H14	119.7
C6—C5—H5	119.7	O3—C15—C14	116.20 (16)
C5—C6—C1	117.15 (15)	O3—C15—C16	124.10 (16)
C5—C6—N1	122.11 (14)	C14—C15—C16	119.69 (16)
C1—C6—N1	120.68 (14)	C17—C16—C15	119.06 (16)
N1—C7—C8	111.59 (14)	C17—C16—H16	120.5
N1—C7—H7A	109.3	C15—C16—H16	120.5
C8—C7—H7A	109.3	C16—C17—C12	122.16 (16)
N1—C7—H7B	109.3	C16—C17—H17	118.9
C8—C7—H7B	109.3	C12—C17—H17	118.9
H7A—C7—H7B	108	O3—C18—H13A	109.5
N2—C8—C7	110.40 (13)	O3—C18—H13B	109.5
N2—C8—H8A	109.6	H13A—C18—H13B	109.5
C7—C8—H8A	109.6	O3—C18—H13C	109.5
N2—C8—H8B	109.6	H13A—C18—H13C	109.5
C7—C8—H8B	109.6	H13B—C18—H13C	109.5
H8A—C8—H8B	108.1	C6—N1—C10	115.62 (12)
N2—C9—C10	110.34 (14)	C6—N1—C7	114.90 (12)
N2—C9—H9A	109.6	C10—N1—C7	111.53 (12)
C10—C9—H9A	109.6	C9—N2—C8	109.67 (13)
N2—C9—H9B	109.6	C9—N2—HN1	108.7 (13)
C10—C9—H9B	109.6	C8—N2—HN1	110.6 (13)
H9A—C9—H9B	108.1	C9—N2—HN2	110.0 (13)
N1—C10—C9	112.30 (13)	C8—N2—HN2	113.2 (13)
N1—C10—H10A	109.1	HN1—N2—HN2	104.5 (18)
C9—C10—H10A	109.1	C15—O3—C18	117.68 (16)
N1—C10—H10B	109.1	HW1—OW1—HW2	106 (2)
			
C6—C1—C2—C3	−0.7 (3)	C13—C14—C15—C16	−1.1 (3)
C1—C2—C3—C4	1.2 (3)	O3—C15—C16—C17	−179.35 (16)
C2—C3—C4—C5	−0.9 (3)	C14—C15—C16—C17	0.7 (3)
C3—C4—C5—C6	0.0 (3)	C15—C16—C17—C12	0.5 (3)
C4—C5—C6—C1	0.6 (3)	C13—C12—C17—C16	−1.4 (2)
C4—C5—C6—N1	−176.76 (18)	C11—C12—C17—C16	178.68 (15)
C2—C1—C6—C5	−0.2 (3)	C5—C6—N1—C10	−7.1 (2)
C2—C1—C6—N1	177.14 (18)	C1—C6—N1—C10	175.65 (16)
N1—C7—C8—N2	−57.13 (19)	C5—C6—N1—C7	−139.28 (17)
N2—C9—C10—N1	55.4 (2)	C1—C6—N1—C7	43.5 (2)
O1—C11—C12—C17	177.14 (17)	C9—C10—N1—C6	172.93 (14)
O2—C11—C12—C17	−3.0 (2)	C9—C10—N1—C7	−53.34 (19)
O1—C11—C12—C13	−2.8 (2)	C8—C7—N1—C6	−171.86 (13)
O2—C11—C12—C13	177.07 (16)	C8—C7—N1—C10	54.06 (18)
C17—C12—C13—C14	1.0 (2)	C10—C9—N2—C8	−57.61 (19)
C11—C12—C13—C14	−179.05 (15)	C7—C8—N2—C9	58.76 (19)
C12—C13—C14—C15	0.2 (3)	C14—C15—O3—C18	178.00 (19)
C13—C14—C15—O3	178.99 (15)	C16—C15—O3—C18	−1.9 (3)

### 4-Phenylpiperazin-1-ium 4-methoxybenzoate monohydrate (II) Hydrogen-bond geometry (Å, º)

*Cg*3 is the centroid of the C12–C17 benzene ring.

**Table:**

*D*—H···*A*	*D*—H	H···*A*	*D*···*A*	*D*—H···*A*
N2—H*N*1···O*W*1^i^	0.93 (2)	1.91 (2)	2.815 (2)	166 (2)
O*W*1—H*W*1···O1^ii^	0.84 (2)	1.80 (2)	2.633 (2)	175 (2)
N2—H*N*2···O2	0.93 (2)	1.81 (2)	2.7350 (19)	176 (2)
O*W*1—H*W*2···O2^iii^	0.85 (2)	1.96 (2)	2.7876 (19)	168 (2)
C8—H8*B*···O*W*1^ii^	0.97	2.53	3.331 (2)	140
C1—H1···*Cg*3^ii^	0.93	2.76	3.549 (2)	144
C5—H5···*Cg*3^iv^	0.93	2.86	3.625 (2)	140

Symmetry codes: (i) *x*, *y*−1, *z*; (ii) −*x*, −*y*+1, −*z*+1; (iii) −*x*+1, −*y*+1, −*z*+1; (iv) −*x*+1, −*y*, −*z*+1.

### 4-Phenylpiperazin-1-ium 4-methylbenzoate monohydrate (III) Crystal data

**Table:**

C_10_H_15_N_2_^+^·C_8_H_7_O_2_^−^·H_2_O	*Z* = 2
*M**_r_* = 316.39	*F*(000) = 340
Triclinic, *P*1	*D*_x_ = 1.233 Mg m^−^^3^
Hall symbol: -P 1	Mo *K*α radiation, λ = 0.71073 Å
*a* = 6.1175 (5) Å	Cell parameters from 2877 reflections
*b* = 7.6225 (7) Å	θ = 3.0–27.8°
*c* = 18.452 (1) Å	µ = 0.08 mm^−^^1^
α = 97.421 (9)°	*T* = 293 K
β = 90.403 (8)°	Plate, colourless
γ = 92.405 (8)°	0.5 × 0.4 × 0.08 mm
*V* = 852.40 (12) Å^3^	

### 4-Phenylpiperazin-1-ium 4-methylbenzoate monohydrate (III) Data collection

**Table:**

Oxford Diffraction Xcalibur diffractometer	2248 reflections with *I* > 2σ(*I*)
ω scans	*R*_int_ = 0.013
Absorption correction: multi-scan (CrysAlis RED; Oxford Diffraction, 2009)	θ_max_ = 25.4°, θ_min_ = 3.1°
*T*_min_ = 0.837, *T*_max_ = 1.000	*h* = −7→7
5354 measured reflections	*k* = −7→9
3126 independent reflections	*l* = −22→22

### 4-Phenylpiperazin-1-ium 4-methylbenzoate monohydrate (III) Refinement

**Table:**

Refinement on *F*^2^	Secondary atom site location: structure-invariant direct methods
Least-squares matrix: full	Hydrogen site location: mixed
*R*[*F*^2^ > 2σ(*F*^2^)] = 0.046	H atoms treated by a mixture of independent and constrained refinement
*wR*(*F*^2^) = 0.128	*w* = 1/[σ^2^(*F*_o_^2^) + (0.0582*P*)^2^ + 0.2086*P*] where *P* = (*F*_o_^2^ + 2*F*_c_^2^)/3
*S* = 1.03	(Δ/σ)_max_ < 0.001
3118 reflections	Δρ_max_ = 0.16 e Å^−^^3^
226 parameters	Δρ_min_ = −0.16 e Å^−^^3^
4 restraints	Extinction correction: SHELXL2018/3 (Sheldrick 2015b), Fc^*^=kFc[1+0.001xFc^2^λ^3^/sin(2θ)]^-1/4^
0 constraints	Extinction coefficient: 0.011 (3)
Primary atom site location: structure-invariant direct methods	

### 4-Phenylpiperazin-1-ium 4-methylbenzoate monohydrate (III) Special details

**Table:**

Geometry. All esds (except the esd in the dihedral angle between two l.s. planes) are estimated using the full covariance matrix. The cell esds are taken into account individually in the estimation of esds in distances, angles and torsion angles; correlations between esds in cell parameters are only used when they are defined by crystal symmetry. An approximate (isotropic) treatment of cell esds is used for estimating esds involving l.s. planes.

### 4-Phenylpiperazin-1-ium 4-methylbenzoate monohydrate (III) Fractional atomic coordinates and isotropic or equivalent isotropic displacement parameters (Å^2^)

**Table:**

	*x*	*y*	*z*	*U*_iso_*/*U*_eq_	
C1	0.5691 (4)	0.8192 (4)	0.17484 (12)	0.0759 (7)	
H1	0.680156	0.871649	0.206032	0.091*	
C2	0.5967 (5)	0.8079 (4)	0.10029 (13)	0.0979 (9)	
H2	0.725499	0.854112	0.082348	0.117*	
C3	0.4416 (5)	0.7314 (4)	0.05273 (13)	0.0932 (8)	
H3	0.462879	0.722711	0.002561	0.112*	
C4	0.2536 (5)	0.6676 (4)	0.08016 (13)	0.0966 (9)	
H4	0.144014	0.6157	0.048248	0.116*	
C5	0.2219 (4)	0.6782 (3)	0.15497 (11)	0.0776 (7)	
H5	0.091389	0.633226	0.172178	0.093*	
C6	0.3793 (3)	0.7539 (2)	0.20421 (9)	0.0472 (4)	
C7	0.5393 (3)	0.8071 (3)	0.32686 (9)	0.0497 (4)	
H7A	0.626347	0.904686	0.31147	0.06*	
H7B	0.626834	0.703106	0.320724	0.06*	
C8	0.4853 (3)	0.8506 (3)	0.40599 (10)	0.0534 (5)	
H8A	0.619196	0.865746	0.435007	0.064*	
H8B	0.410139	0.960943	0.413335	0.064*	
C9	0.1390 (3)	0.6889 (3)	0.38632 (10)	0.0554 (5)	
H9A	0.062182	0.798326	0.394853	0.067*	
H9B	0.04531	0.594989	0.401599	0.067*	
C10	0.1858 (3)	0.6476 (3)	0.30616 (9)	0.0502 (5)	
H10A	0.241603	0.529612	0.296889	0.06*	
H10B	0.050244	0.647749	0.278608	0.06*	
C11	0.3129 (3)	0.7088 (2)	0.62385 (9)	0.0445 (4)	
C12	0.2060 (3)	0.7367 (2)	0.69737 (9)	0.0413 (4)	
C13	0.2989 (3)	0.6749 (3)	0.75734 (10)	0.0535 (5)	
H13	0.428845	0.615675	0.752062	0.064*	
C14	0.2008 (4)	0.7003 (3)	0.82503 (11)	0.0656 (6)	
H14	0.265661	0.657329	0.864556	0.079*	
C15	0.0081 (4)	0.7885 (3)	0.83492 (11)	0.0611 (5)	
C16	−0.0837 (3)	0.8490 (3)	0.77515 (11)	0.0583 (5)	
H16	−0.214079	0.907628	0.780509	0.07*	
C17	0.0129 (3)	0.8250 (2)	0.70719 (10)	0.0484 (4)	
H17	−0.052163	0.868519	0.667839	0.058*	
C18	−0.0996 (5)	0.8177 (4)	0.90877 (13)	0.0968 (9)	
H18A	−0.171921	0.927853	0.913714	0.145*	
H18B	0.009588	0.821099	0.946613	0.145*	
H18C	−0.204829	0.722525	0.912877	0.145*	
N1	0.3441 (2)	0.77371 (19)	0.28025 (7)	0.0434 (4)	
N2	0.3444 (2)	0.7069 (2)	0.43010 (8)	0.0487 (4)	
O1	0.4844 (2)	0.62506 (19)	0.61816 (7)	0.0632 (4)	
O2	0.2244 (2)	0.77468 (19)	0.57233 (7)	0.0601 (4)	
OW1	0.7937 (3)	0.7241 (4)	0.52207 (11)	0.1232 (9)	
HW1	0.926 (4)	0.748 (5)	0.542 (2)	0.177 (17)*	
HW2	0.702 (4)	0.704 (4)	0.5539 (13)	0.119 (11)*	
HN1	0.415 (3)	0.605 (2)	0.4225 (11)	0.061 (6)*	
HN2	0.310 (3)	0.731 (3)	0.4786 (9)	0.065 (6)*	

### 4-Phenylpiperazin-1-ium 4-methylbenzoate monohydrate (III) Atomic displacement parameters (Å^2^)

**Table:**

	*U*^11^	*U*^22^	*U*^33^	*U*^12^	*U*^13^	*U*^23^
C1	0.0620 (13)	0.114 (2)	0.0494 (12)	−0.0113 (13)	0.0106 (10)	0.0080 (12)
C2	0.0883 (18)	0.148 (3)	0.0556 (15)	−0.0138 (18)	0.0233 (13)	0.0120 (15)
C3	0.123 (2)	0.109 (2)	0.0456 (13)	−0.0055 (18)	0.0201 (15)	0.0043 (13)
C4	0.130 (2)	0.106 (2)	0.0480 (13)	−0.0340 (18)	−0.0161 (14)	0.0036 (13)
C5	0.0895 (16)	0.0936 (17)	0.0466 (12)	−0.0337 (13)	−0.0042 (11)	0.0105 (11)
C6	0.0561 (11)	0.0456 (10)	0.0397 (9)	0.0045 (8)	0.0038 (8)	0.0042 (7)
C7	0.0473 (10)	0.0550 (11)	0.0456 (10)	−0.0123 (8)	0.0008 (8)	0.0070 (8)
C8	0.0581 (11)	0.0569 (12)	0.0440 (10)	−0.0065 (9)	−0.0031 (8)	0.0045 (8)
C9	0.0439 (10)	0.0734 (13)	0.0494 (11)	−0.0025 (9)	0.0087 (8)	0.0105 (9)
C10	0.0383 (9)	0.0668 (12)	0.0446 (10)	−0.0056 (8)	0.0005 (7)	0.0064 (8)
C11	0.0429 (10)	0.0454 (10)	0.0432 (10)	−0.0062 (8)	0.0042 (7)	0.0012 (7)
C12	0.0413 (9)	0.0376 (9)	0.0439 (9)	−0.0047 (7)	0.0046 (7)	0.0025 (7)
C13	0.0525 (11)	0.0601 (12)	0.0479 (11)	0.0081 (9)	0.0012 (8)	0.0054 (9)
C14	0.0798 (15)	0.0739 (14)	0.0439 (11)	0.0046 (11)	−0.0013 (10)	0.0111 (9)
C15	0.0742 (14)	0.0597 (12)	0.0479 (11)	−0.0009 (10)	0.0167 (10)	0.0012 (9)
C16	0.0577 (12)	0.0554 (12)	0.0614 (12)	0.0098 (9)	0.0173 (9)	0.0025 (9)
C17	0.0525 (10)	0.0458 (10)	0.0476 (10)	0.0047 (8)	0.0056 (8)	0.0072 (8)
C18	0.125 (2)	0.108 (2)	0.0567 (14)	0.0110 (17)	0.0378 (15)	0.0048 (13)
N1	0.0436 (8)	0.0487 (8)	0.0379 (8)	−0.0025 (6)	0.0016 (6)	0.0068 (6)
N2	0.0519 (9)	0.0577 (10)	0.0371 (8)	0.0057 (8)	0.0054 (7)	0.0068 (7)
O1	0.0546 (8)	0.0720 (9)	0.0633 (9)	0.0149 (7)	0.0170 (6)	0.0047 (7)
O2	0.0557 (8)	0.0833 (10)	0.0426 (7)	0.0032 (7)	0.0096 (6)	0.0120 (7)
OW1	0.0607 (12)	0.250 (3)	0.0691 (12)	0.0102 (15)	0.0088 (10)	0.0577 (15)

### 4-Phenylpiperazin-1-ium 4-methylbenzoate monohydrate (III) Geometric parameters (Å, º)

**Table:**

C1—C2	1.379 (3)	C10—H10A	0.97
C1—C6	1.385 (3)	C10—H10B	0.97
C1—H1	0.93	C11—O1	1.249 (2)
C2—C3	1.349 (4)	C11—O2	1.260 (2)
C2—H2	0.93	C11—C12	1.504 (2)
C3—C4	1.356 (4)	C12—C13	1.385 (2)
C3—H3	0.93	C12—C17	1.386 (2)
C4—C5	1.388 (3)	C13—C14	1.384 (3)
C4—H4	0.93	C13—H13	0.93
C5—C6	1.375 (3)	C14—C15	1.383 (3)
C5—H5	0.93	C14—H14	0.93
C6—N1	1.411 (2)	C15—C16	1.374 (3)
C7—N1	1.461 (2)	C15—C18	1.512 (3)
C7—C8	1.497 (2)	C16—C17	1.384 (2)
C7—H7A	0.97	C16—H16	0.93
C7—H7B	0.97	C17—H17	0.93
C8—N2	1.481 (2)	C18—H18A	0.96
C8—H8A	0.97	C18—H18B	0.96
C8—H8B	0.97	C18—H18C	0.96
C9—N2	1.481 (2)	N2—HN1	0.900 (15)
C9—C10	1.504 (2)	N2—HN2	0.918 (15)
C9—H9A	0.97	OW1—HW1	0.886 (19)
C9—H9B	0.97	OW1—HW2	0.839 (18)
C10—N1	1.462 (2)		
			
C2—C1—C6	121.3 (2)	C9—C10—H10B	109
C2—C1—H1	119.3	H10A—C10—H10B	107.8
C6—C1—H1	119.3	O1—C11—O2	124.44 (16)
C3—C2—C1	121.6 (2)	O1—C11—C12	118.17 (16)
C3—C2—H2	119.2	O2—C11—C12	117.37 (15)
C1—C2—H2	119.2	C13—C12—C17	118.11 (16)
C2—C3—C4	118.1 (2)	C13—C12—C11	120.69 (15)
C2—C3—H3	120.9	C17—C12—C11	121.19 (15)
C4—C3—H3	120.9	C14—C13—C12	120.81 (17)
C3—C4—C5	121.2 (2)	C14—C13—H13	119.6
C3—C4—H4	119.4	C12—C13—H13	119.6
C5—C4—H4	119.4	C15—C14—C13	121.14 (19)
C6—C5—C4	121.4 (2)	C15—C14—H14	119.4
C6—C5—H5	119.3	C13—C14—H14	119.4
C4—C5—H5	119.3	C16—C15—C14	117.78 (17)
C5—C6—C1	116.25 (18)	C16—C15—C18	120.7 (2)
C5—C6—N1	121.84 (18)	C14—C15—C18	121.5 (2)
C1—C6—N1	121.81 (17)	C15—C16—C17	121.68 (18)
N1—C7—C8	112.51 (14)	C15—C16—H16	119.2
N1—C7—H7A	109.1	C17—C16—H16	119.2
C8—C7—H7A	109.1	C16—C17—C12	120.47 (17)
N1—C7—H7B	109.1	C16—C17—H17	119.8
C8—C7—H7B	109.1	C12—C17—H17	119.8
H7A—C7—H7B	107.8	C15—C18—H18A	109.5
N2—C8—C7	110.19 (15)	C15—C18—H18B	109.5
N2—C8—H8A	109.6	H18A—C18—H18B	109.5
C7—C8—H8A	109.6	C15—C18—H18C	109.5
N2—C8—H8B	109.6	H18A—C18—H18C	109.5
C7—C8—H8B	109.6	H18B—C18—H18C	109.5
H8A—C8—H8B	108.1	C6—N1—C7	116.19 (14)
N2—C9—C10	110.84 (14)	C6—N1—C10	116.09 (14)
N2—C9—H9A	109.5	C7—N1—C10	113.11 (13)
C10—C9—H9A	109.5	C8—N2—C9	108.70 (15)
N2—C9—H9B	109.5	C8—N2—HN1	108.9 (13)
C10—C9—H9B	109.5	C9—N2—HN1	109.5 (13)
H9A—C9—H9B	108.1	C8—N2—HN2	111.2 (13)
N1—C10—C9	112.96 (15)	C9—N2—HN2	108.7 (13)
N1—C10—H10A	109	HN1—N2—HN2	109.8 (18)
C9—C10—H10A	109	HW1—OW1—HW2	111 (3)
N1—C10—H10B	109		
			
C6—C1—C2—C3	−0.7 (5)	C13—C14—C15—C16	0.4 (3)
C1—C2—C3—C4	1.1 (5)	C13—C14—C15—C18	−179.7 (2)
C2—C3—C4—C5	−0.8 (5)	C14—C15—C16—C17	−0.6 (3)
C3—C4—C5—C6	0.1 (4)	C18—C15—C16—C17	179.5 (2)
C4—C5—C6—C1	0.4 (4)	C15—C16—C17—C12	0.7 (3)
C4—C5—C6—N1	176.6 (2)	C13—C12—C17—C16	−0.5 (3)
C2—C1—C6—C5	−0.1 (4)	C11—C12—C17—C16	179.76 (17)
C2—C1—C6—N1	−176.3 (2)	C5—C6—N1—C7	162.90 (19)
N1—C7—C8—N2	56.4 (2)	C1—C6—N1—C7	−21.0 (3)
N2—C9—C10—N1	−53.3 (2)	C5—C6—N1—C10	26.2 (3)
O1—C11—C12—C13	1.6 (2)	C1—C6—N1—C10	−157.72 (19)
O2—C11—C12—C13	−177.16 (17)	C8—C7—N1—C6	172.21 (15)
O1—C11—C12—C17	−178.61 (16)	C8—C7—N1—C10	−49.8 (2)
O2—C11—C12—C17	2.6 (2)	C9—C10—N1—C6	−173.76 (14)
C17—C12—C13—C14	0.3 (3)	C9—C10—N1—C7	48.2 (2)
C11—C12—C13—C14	−179.94 (18)	C7—C8—N2—C9	−60.59 (19)
C12—C13—C14—C15	−0.3 (3)	C10—C9—N2—C8	59.1 (2)

### 4-Phenylpiperazin-1-ium 4-methylbenzoate monohydrate (III) Hydrogen-bond geometry (Å, º)

**Table:**

*D*—H···*A*	*D*—H	H···*A*	*D*···*A*	*D*—H···*A*
O*W*1—H*W*1···O2^i^	0.89 (3)	1.90 (3)	2.782 (2)	171 (4)
O*W*1—H*W*2···O1	0.84 (2)	1.92 (3)	2.751 (2)	172 (3)
N2—H*N*1···O1^ii^	0.90 (2)	1.94 (2)	2.819 (2)	164 (2)
N2—H*N*2···O2	0.92 (2)	1.80 (2)	2.7207 (19)	176 (2)
C8—H8*A*···O*W*1	0.97	2.33	3.116 (3)	138

Symmetry codes: (i) *x*+1, *y*, *z*; (ii) −*x*+1, −*y*+1, −*z*+1.

### 4-Phenylpiperazin-1-ium trifluoroacetate 0.123-hydrate (IV) Crystal data

**Table:**

C_10_H_15_N_2_^+^·C_2_F_3_O_2_^−^·0.123H_2_O	*Z* = 4
*M**_r_* = 278.47	*F*(000) = 580.9
Triclinic, *P*1	*D*_x_ = 1.371 Mg m^−^^3^
Hall symbol: -P 1	Mo *K*α radiation, λ = 0.71073 Å
*a* = 9.6544 (6) Å	Cell parameters from 3827 reflections
*b* = 9.9029 (6) Å	θ = 2.6–27.7°
*c* = 15.2090 (9) Å	µ = 0.12 mm^−^^1^
α = 79.621 (6)°	*T* = 293 K
β = 86.579 (6)°	Prism, colourless
γ = 70.603 (6)°	0.48 × 0.48 × 0.36 mm
*V* = 1349.10 (15) Å^3^	

### 4-Phenylpiperazin-1-ium trifluoroacetate 0.123-hydrate (IV) Data collection

**Table:**

Oxford Diffraction Xcalibur diffractometer	2777 reflections with *I* > 2σ(*I*)
ω scans	*R*_int_ = 0.014
Absorption correction: multi-scan (CrysAlis RED; Oxford Diffraction, 2009)	θ_max_ = 25.3°, θ_min_ = 2.6°
*T*_min_ = 0.724, *T*_max_ = 1.000	*h* = −11→11
9220 measured reflections	*k* = −11→11
4940 independent reflections	*l* = −18→18

### 4-Phenylpiperazin-1-ium trifluoroacetate 0.123-hydrate (IV) Refinement

**Table:**

Refinement on *F*^2^	Primary atom site location: structure-invariant direct methods
Least-squares matrix: full	Secondary atom site location: structure-invariant direct methods
*R*[*F*^2^ > 2σ(*F*^2^)] = 0.070	Hydrogen site location: mixed
*wR*(*F*^2^) = 0.235	H-atom parameters constrained
*S* = 1.07	*w* = 1/[σ^2^(*F*_o_^2^) + (0.1128*P*)^2^ + 0.3819*P*] where *P* = (*F*_o_^2^ + 2*F*_c_^2^)/3
4927 reflections	(Δ/σ)_max_ < 0.001
375 parameters	Δρ_max_ = 0.42 e Å^−^^3^
4 restraints	Δρ_min_ = −0.28 e Å^−^^3^
0 constraints	

### 4-Phenylpiperazin-1-ium trifluoroacetate 0.123-hydrate (IV) Special details

**Table:**

Geometry. All esds (except the esd in the dihedral angle between two l.s. planes) are estimated using the full covariance matrix. The cell esds are taken into account individually in the estimation of esds in distances, angles and torsion angles; correlations between esds in cell parameters are only used when they are defined by crystal symmetry. An approximate (isotropic) treatment of cell esds is used for estimating esds involving l.s. planes.

### 4-Phenylpiperazin-1-ium trifluoroacetate 0.123-hydrate (IV) Fractional atomic coordinates and isotropic or equivalent isotropic displacement parameters (Å^2^)

**Table:**

	*x*	*y*	*z*	*U*_iso_*/*U*_eq_	Occ. (<1)
C1	0.2559 (3)	0.7294 (3)	0.51366 (18)	0.0565 (7)	
C2	0.1392 (4)	0.7473 (3)	0.5737 (2)	0.0733 (9)	
H2	0.043765	0.775704	0.552306	0.088*	
C3	0.1634 (5)	0.7235 (4)	0.6648 (3)	0.0913 (12)	
H3	0.083976	0.737161	0.703837	0.11*	
C4	0.3019 (6)	0.6803 (4)	0.6979 (2)	0.0945 (12)	
H4	0.317436	0.664341	0.759246	0.113*	
C5	0.4172 (5)	0.6606 (4)	0.6405 (3)	0.0939 (11)	
H5	0.512209	0.630205	0.66299	0.113*	
C6	0.3958 (4)	0.6850 (4)	0.5497 (2)	0.0746 (9)	
H6	0.476639	0.671553	0.511724	0.089*	
C7	0.3582 (4)	0.7522 (4)	0.3646 (2)	0.0748 (9)	
H7A	0.439678	0.665346	0.385364	0.09*	
H7B	0.386552	0.835603	0.369838	0.09*	
C8	0.3289 (5)	0.7565 (4)	0.2686 (2)	0.0924 (11)	
H8A	0.41407	0.762586	0.232966	0.111*	
H8B	0.311597	0.667892	0.261853	0.111*	
C9	0.0709 (5)	0.8815 (5)	0.2926 (3)	0.1078 (14)	
H9A	0.044836	0.796648	0.287542	0.129*	
H9B	−0.01211	0.967225	0.272441	0.129*	
C10	0.1038 (4)	0.8774 (5)	0.3876 (2)	0.0933 (12)	
H10A	0.121556	0.966325	0.393067	0.112*	
H10B	0.019188	0.872467	0.424128	0.112*	
C11	0.7162 (3)	0.7837 (3)	0.34093 (18)	0.0568 (7)	
C12	0.6756 (3)	0.9091 (3)	0.3787 (2)	0.0682 (8)	
H12	0.637995	0.999179	0.342289	0.082*	
C13	0.6902 (4)	0.9023 (4)	0.4692 (2)	0.0777 (9)	
H13	0.660653	0.987687	0.492938	0.093*	
C14	0.7475 (4)	0.7719 (5)	0.5247 (2)	0.0786 (10)	
H14	0.758672	0.767891	0.585529	0.094*	
C15	0.7878 (4)	0.6476 (4)	0.4884 (2)	0.0856 (10)	
H15	0.826468	0.558096	0.525259	0.103*	
C16	0.7723 (4)	0.6527 (4)	0.3984 (2)	0.0760 (9)	
H16	0.799969	0.566471	0.375625	0.091*	
C17	0.8021 (5)	0.6701 (4)	0.2099 (2)	0.0988 (13)	
H17A	0.807268	0.577457	0.246337	0.119*	
H17B	0.898326	0.680875	0.21027	0.119*	
C18	0.7624 (6)	0.6706 (5)	0.1152 (3)	0.1280 (18)	
H18A	0.837937	0.594874	0.090434	0.154*	
H18B	0.670336	0.65126	0.114843	0.154*	
C19	0.6330 (5)	0.9270 (5)	0.0978 (2)	0.0940 (11)	
H19A	0.540215	0.909315	0.096567	0.113*	
H19B	0.622668	1.021169	0.061705	0.113*	
C20	0.6691 (4)	0.9279 (4)	0.1919 (2)	0.0773 (9)	
H20A	0.756179	0.95605	0.191813	0.093*	
H20B	0.58882	0.999996	0.216184	0.093*	
C21	0.2670 (4)	0.2041 (4)	0.1833 (2)	0.0776 (9)	
C22	0.2420 (8)	0.3636 (6)	0.1772 (3)	0.1240 (19)	0.736 (3)
C22'	0.2420 (8)	0.3636 (6)	0.1772 (3)	0.1240 (19)	0.264 (3)
C23	0.1345 (7)	0.8145 (4)	0.0390 (2)	0.0940 (13)	
C24	0.1356 (7)	0.7597 (5)	−0.0474 (3)	0.1064 (14)	0.736 (3)
C24'	0.1356 (7)	0.7597 (5)	−0.0474 (3)	0.1064 (14)	0.264 (3)
N1	0.2321 (3)	0.7528 (2)	0.42089 (15)	0.0596 (6)	
N2	0.1985 (4)	0.8842 (3)	0.23622 (18)	0.0888 (9)	
N3	0.6944 (3)	0.7878 (3)	0.24907 (15)	0.0687 (7)	
N4	0.7485 (4)	0.8143 (4)	0.05995 (19)	0.1087 (12)	
O1	0.2367 (4)	0.1448 (3)	0.25394 (17)	0.1168 (10)	
O2	0.3178 (4)	0.1492 (4)	0.11854 (18)	0.1323 (12)	
O3	0.2462 (4)	0.8383 (4)	0.05509 (19)	0.1252 (11)	
O4	0.0222 (4)	0.8316 (3)	0.08423 (17)	0.1113 (10)	
F1	0.3669 (7)	0.3949 (7)	0.1721 (5)	0.202 (3)	0.736 (3)
F2	0.1775 (9)	0.4242 (5)	0.2477 (4)	0.184 (3)	0.736 (3)
F3	0.1706 (8)	0.4421 (5)	0.1039 (4)	0.179 (3)	0.736 (3)
F1'	0.302 (2)	0.407 (2)	0.0998 (15)	0.202 (3)	0.264 (3)
F2'	0.282 (3)	0.3950 (17)	0.2311 (15)	0.184 (3)	0.264 (3)
F3'	0.102 (2)	0.4303 (16)	0.1642 (11)	0.179 (3)	0.264 (3)
F4	0.2609 (7)	0.6549 (8)	−0.0615 (4)	0.175 (2)	0.736 (3)
F5	0.1187 (10)	0.8524 (4)	−0.1159 (2)	0.181 (3)	0.736 (3)
F6	0.0480 (7)	0.6817 (8)	−0.0460 (4)	0.153 (2)	0.736 (3)
F4'	0.238 (2)	0.789 (2)	−0.1015 (13)	0.175 (2)	0.264 (3)
F5'	0.157 (4)	0.6370 (19)	−0.0412 (9)	0.181 (3)	0.264 (3)
F6'	−0.0072 (19)	0.8256 (19)	−0.0913 (10)	0.153 (2)	0.264 (3)
OW1	0.5122 (12)	0.6320 (14)	0.0361 (10)	0.150 (8)	0.245 (10)
H21	0.207318	0.969376	0.238227	0.18*	
H22	0.187596	0.886717	0.179696	0.18*	
H41	0.826598	0.838953	0.053835	0.18*	
H42	0.722096	0.810784	0.005806	0.18*	
HW1A	0.572085	0.618747	−0.007306	0.225*	0.245 (10)
HW1B	0.441675	0.707638	0.015874	0.225*	0.245 (10)

### 4-Phenylpiperazin-1-ium trifluoroacetate 0.123-hydrate (IV) Atomic displacement parameters (Å^2^)

**Table:**

	*U*^11^	*U*^22^	*U*^33^	*U*^12^	*U*^13^	*U*^23^
C1	0.065 (2)	0.0490 (15)	0.0531 (16)	−0.0184 (13)	0.0022 (14)	−0.0040 (12)
C2	0.075 (2)	0.069 (2)	0.068 (2)	−0.0195 (16)	0.0111 (17)	−0.0051 (15)
C3	0.116 (3)	0.074 (2)	0.075 (2)	−0.025 (2)	0.033 (2)	−0.0120 (18)
C4	0.141 (4)	0.079 (2)	0.056 (2)	−0.028 (2)	−0.001 (2)	−0.0089 (17)
C5	0.107 (3)	0.098 (3)	0.072 (2)	−0.025 (2)	−0.026 (2)	−0.0087 (19)
C6	0.064 (2)	0.089 (2)	0.066 (2)	−0.0204 (17)	−0.0069 (16)	−0.0074 (16)
C7	0.073 (2)	0.086 (2)	0.0618 (18)	−0.0228 (17)	0.0105 (16)	−0.0132 (16)
C8	0.122 (3)	0.098 (3)	0.062 (2)	−0.039 (2)	0.010 (2)	−0.0215 (19)
C9	0.099 (3)	0.139 (4)	0.079 (3)	−0.044 (3)	−0.030 (2)	0.017 (2)
C10	0.065 (2)	0.128 (3)	0.063 (2)	−0.010 (2)	−0.0084 (17)	0.0067 (19)
C11	0.0597 (18)	0.0649 (18)	0.0512 (15)	−0.0254 (14)	0.0087 (13)	−0.0168 (13)
C12	0.086 (2)	0.0636 (18)	0.0605 (17)	−0.0280 (16)	0.0031 (15)	−0.0183 (14)
C13	0.089 (2)	0.093 (2)	0.066 (2)	−0.0390 (19)	0.0122 (18)	−0.0374 (19)
C14	0.076 (2)	0.119 (3)	0.0513 (17)	−0.044 (2)	0.0031 (16)	−0.019 (2)
C15	0.092 (3)	0.093 (3)	0.063 (2)	−0.024 (2)	−0.0075 (18)	−0.0006 (18)
C16	0.091 (2)	0.065 (2)	0.066 (2)	−0.0172 (16)	0.0037 (17)	−0.0140 (15)
C17	0.167 (4)	0.071 (2)	0.064 (2)	−0.042 (2)	0.028 (2)	−0.0283 (17)
C18	0.221 (6)	0.115 (4)	0.074 (3)	−0.078 (4)	0.039 (3)	−0.050 (2)
C19	0.118 (3)	0.118 (3)	0.0538 (19)	−0.051 (2)	0.0026 (19)	−0.0131 (19)
C20	0.094 (3)	0.076 (2)	0.0602 (18)	−0.0257 (18)	−0.0026 (17)	−0.0121 (16)
C21	0.102 (3)	0.086 (2)	0.0458 (17)	−0.0309 (19)	−0.0063 (17)	−0.0135 (16)
C22	0.194 (6)	0.122 (4)	0.068 (3)	−0.074 (4)	−0.044 (3)	0.011 (3)
C22'	0.194 (6)	0.122 (4)	0.068 (3)	−0.074 (4)	−0.044 (3)	0.011 (3)
C23	0.159 (4)	0.066 (2)	0.052 (2)	−0.032 (2)	0.011 (3)	−0.0113 (16)
C24	0.172 (5)	0.084 (3)	0.070 (2)	−0.047 (3)	0.028 (3)	−0.028 (2)
C24'	0.172 (5)	0.084 (3)	0.070 (2)	−0.047 (3)	0.028 (3)	−0.028 (2)
N1	0.0591 (15)	0.0674 (15)	0.0519 (13)	−0.0234 (12)	−0.0030 (11)	−0.0028 (11)
N2	0.136 (3)	0.082 (2)	0.0530 (15)	−0.0447 (19)	−0.0139 (17)	−0.0015 (13)
N3	0.094 (2)	0.0709 (16)	0.0501 (13)	−0.0357 (14)	0.0087 (12)	−0.0184 (12)
N4	0.162 (3)	0.127 (3)	0.0538 (16)	−0.066 (2)	0.0207 (19)	−0.0303 (18)
O1	0.205 (3)	0.0908 (18)	0.0635 (16)	−0.0618 (19)	0.0128 (17)	−0.0133 (13)
O2	0.163 (3)	0.164 (3)	0.0675 (16)	−0.038 (2)	0.0098 (17)	−0.0474 (18)
O3	0.161 (3)	0.157 (3)	0.0719 (18)	−0.062 (3)	0.0133 (19)	−0.0395 (18)
O4	0.154 (3)	0.121 (2)	0.0633 (15)	−0.0477 (19)	0.0290 (17)	−0.0313 (14)
F1	0.249 (6)	0.248 (6)	0.198 (6)	−0.196 (5)	−0.015 (4)	−0.040 (4)
F2	0.289 (8)	0.078 (3)	0.159 (4)	−0.016 (4)	0.037 (5)	−0.053 (2)
F3	0.278 (7)	0.117 (3)	0.142 (4)	−0.095 (3)	−0.104 (5)	0.068 (3)
F1'	0.249 (6)	0.248 (6)	0.198 (6)	−0.196 (5)	−0.015 (4)	−0.040 (4)
F2'	0.289 (8)	0.078 (3)	0.159 (4)	−0.016 (4)	0.037 (5)	−0.053 (2)
F3'	0.278 (7)	0.117 (3)	0.142 (4)	−0.095 (3)	−0.104 (5)	0.068 (3)
F4	0.227 (5)	0.149 (4)	0.137 (4)	−0.024 (4)	0.046 (3)	−0.082 (4)
F5	0.411 (11)	0.084 (2)	0.0504 (17)	−0.087 (4)	−0.014 (3)	0.0007 (16)
F6	0.226 (5)	0.152 (4)	0.118 (3)	−0.092 (4)	0.009 (3)	−0.061 (3)
F4'	0.227 (5)	0.149 (4)	0.137 (4)	−0.024 (4)	0.046 (3)	−0.082 (4)
F5'	0.411 (11)	0.084 (2)	0.0504 (17)	−0.087 (4)	−0.014 (3)	0.0007 (16)
F6'	0.226 (5)	0.152 (4)	0.118 (3)	−0.092 (4)	0.009 (3)	−0.061 (3)
OW1	0.082 (10)	0.146 (13)	0.219 (18)	−0.019 (8)	−0.031 (9)	−0.048 (11)

### 4-Phenylpiperazin-1-ium trifluoroacetate 0.123-hydrate (IV) Geometric parameters (Å, º)

**Table:**

C1—C6	1.388 (4)	C17—C18	1.512 (5)
C1—C2	1.392 (4)	C17—H17A	0.97
C1—N1	1.408 (3)	C17—H17B	0.97
C2—C3	1.384 (5)	C18—N4	1.485 (6)
C2—H2	0.93	C18—H18A	0.97
C3—C4	1.359 (6)	C18—H18B	0.97
C3—H3	0.93	C19—N4	1.465 (5)
C4—C5	1.357 (5)	C19—C20	1.495 (4)
C4—H4	0.93	C19—H19A	0.97
C5—C6	1.375 (5)	C19—H19B	0.97
C5—H5	0.93	C20—N3	1.451 (4)
C6—H6	0.93	C20—H20A	0.97
C7—N1	1.445 (4)	C20—H20B	0.97
C7—C8	1.494 (5)	C21—O1	1.197 (4)
C7—H7A	0.97	C21—O2	1.211 (4)
C7—H7B	0.97	C21—C22'	1.502 (7)
C8—N2	1.489 (5)	C21—C22	1.502 (7)
C8—H8A	0.97	C22—F3	1.321 (6)
C8—H8B	0.97	C22—F1	1.337 (7)
C9—N2	1.464 (5)	C22—F2	1.343 (7)
C9—C10	1.488 (5)	C22'—F2'	1.06 (2)
C9—H9A	0.97	C22'—F3'	1.306 (19)
C9—H9B	0.97	C22'—F1'	1.346 (19)
C10—N1	1.466 (4)	C23—O3	1.224 (5)
C10—H10A	0.97	C23—O4	1.228 (5)
C10—H10B	0.97	C23—C24'	1.506 (6)
C11—C16	1.386 (4)	C23—C24	1.506 (6)
C11—C12	1.390 (4)	C24—F5	1.238 (5)
C11—N3	1.417 (3)	C24—F6	1.319 (7)
C12—C13	1.379 (4)	C24—F4	1.342 (7)
C12—H12	0.93	C24'—F5'	1.151 (17)
C13—C14	1.369 (5)	C24'—F4'	1.32 (2)
C13—H13	0.93	C24'—F6'	1.459 (17)
C14—C15	1.368 (5)	N2—H21	0.8818
C14—H14	0.93	N2—H22	0.8672
C15—C16	1.377 (5)	N4—H41	0.8621
C15—H15	0.93	N4—H42	0.8861
C16—H16	0.93	OW1—HW1A	0.8501
C17—N3	1.471 (4)	OW1—HW1B	0.8501
			
C6—C1—C2	116.9 (3)	N4—C18—H18B	109.8
C6—C1—N1	122.0 (3)	C17—C18—H18B	109.8
C2—C1—N1	121.1 (3)	H18A—C18—H18B	108.2
C3—C2—C1	120.8 (3)	N4—C19—C20	110.7 (3)
C3—C2—H2	119.6	N4—C19—H19A	109.5
C1—C2—H2	119.6	C20—C19—H19A	109.5
C4—C3—C2	120.8 (3)	N4—C19—H19B	109.5
C4—C3—H3	119.6	C20—C19—H19B	109.5
C2—C3—H3	119.6	H19A—C19—H19B	108.1
C5—C4—C3	119.2 (4)	N3—C20—C19	112.8 (3)
C5—C4—H4	120.4	N3—C20—H20A	109
C3—C4—H4	120.4	C19—C20—H20A	109
C4—C5—C6	121.0 (4)	N3—C20—H20B	109
C4—C5—H5	119.5	C19—C20—H20B	109
C6—C5—H5	119.5	H20A—C20—H20B	107.8
C5—C6—C1	121.2 (3)	O1—C21—O2	127.2 (4)
C5—C6—H6	119.4	O1—C21—C22'	115.1 (4)
C1—C6—H6	119.4	O2—C21—C22'	117.7 (4)
N1—C7—C8	112.5 (3)	O1—C21—C22	115.1 (4)
N1—C7—H7A	109.1	O2—C21—C22	117.7 (4)
C8—C7—H7A	109.1	F3—C22—F1	103.5 (5)
N1—C7—H7B	109.1	F3—C22—F2	108.8 (6)
C8—C7—H7B	109.1	F1—C22—F2	100.9 (6)
H7A—C7—H7B	107.8	F3—C22—C21	112.7 (4)
N2—C8—C7	110.4 (3)	F1—C22—C21	112.9 (6)
N2—C8—H8A	109.6	F2—C22—C21	116.7 (4)
C7—C8—H8A	109.6	F2'—C22'—F3'	112.1 (15)
N2—C8—H8B	109.6	F2'—C22'—F1'	110.0 (16)
C7—C8—H8B	109.6	F3'—C22'—F1'	103.4 (13)
H8A—C8—H8B	108.1	F2'—C22'—C21	116.5 (11)
N2—C9—C10	110.4 (3)	F3'—C22'—C21	106.9 (7)
N2—C9—H9A	109.6	F1'—C22'—C21	107.0 (10)
C10—C9—H9A	109.6	O3—C23—O4	127.7 (4)
N2—C9—H9B	109.6	O3—C23—C24'	115.5 (5)
C10—C9—H9B	109.6	O4—C23—C24'	116.8 (5)
H9A—C9—H9B	108.1	O3—C23—C24	115.5 (5)
N1—C10—C9	112.1 (3)	O4—C23—C24	116.8 (5)
N1—C10—H10A	109.2	F5—C24—F6	111.9 (6)
C9—C10—H10A	109.2	F5—C24—F4	104.5 (6)
N1—C10—H10B	109.2	F6—C24—F4	96.7 (5)
C9—C10—H10B	109.2	F5—C24—C23	115.4 (4)
H10A—C10—H10B	107.9	F6—C24—C23	113.1 (4)
C16—C11—C12	116.8 (3)	F4—C24—C23	113.6 (5)
C16—C11—N3	121.0 (2)	F5'—C24'—F4'	106.2 (16)
C12—C11—N3	122.0 (3)	F5'—C24'—F6'	103.6 (18)
C13—C12—C11	121.1 (3)	F4'—C24'—F6'	109.6 (11)
C13—C12—H12	119.4	F5'—C24'—C23	116.0 (8)
C11—C12—H12	119.4	F4'—C24'—C23	110.3 (9)
C14—C13—C12	121.1 (3)	F6'—C24'—C23	110.7 (6)
C14—C13—H13	119.4	C1—N1—C7	116.1 (2)
C12—C13—H13	119.4	C1—N1—C10	115.0 (2)
C15—C14—C13	118.3 (3)	C7—N1—C10	110.5 (2)
C15—C14—H14	120.8	C9—N2—C8	110.4 (3)
C13—C14—H14	120.8	C9—N2—H21	104.1
C14—C15—C16	121.2 (3)	C8—N2—H21	115
C14—C15—H15	119.4	C9—N2—H22	115.8
C16—C15—H15	119.4	C8—N2—H22	108.2
C15—C16—C11	121.4 (3)	H21—N2—H22	103.4
C15—C16—H16	119.3	C11—N3—C20	115.9 (2)
C11—C16—H16	119.3	C11—N3—C17	114.7 (3)
N3—C17—C18	111.9 (4)	C20—N3—C17	111.7 (2)
N3—C17—H17A	109.2	C19—N4—C18	109.0 (3)
C18—C17—H17A	109.2	C19—N4—H41	108.2
N3—C17—H17B	109.2	C18—N4—H41	116.5
C18—C17—H17B	109.2	C19—N4—H42	109.5
H17A—C17—H17B	107.9	C18—N4—H42	106.4
N4—C18—C17	109.5 (3)	H41—N4—H42	107.1
N4—C18—H18A	109.8	HW1A—OW1—HW1B	104.5
C17—C18—H18A	109.8		
			
C6—C1—C2—C3	0.8 (4)	O3—C23—C24—F5	−75.6 (8)
N1—C1—C2—C3	179.7 (3)	O4—C23—C24—F5	103.6 (7)
C1—C2—C3—C4	−0.8 (5)	O3—C23—C24—F6	153.8 (6)
C2—C3—C4—C5	0.1 (6)	O4—C23—C24—F6	−26.9 (7)
C3—C4—C5—C6	0.6 (6)	O3—C23—C24—F4	44.9 (7)
C4—C5—C6—C1	−0.5 (6)	O4—C23—C24—F4	−135.8 (6)
C2—C1—C6—C5	−0.2 (5)	O3—C23—C24'—F5'	102 (2)
N1—C1—C6—C5	−179.0 (3)	O4—C23—C24'—F5'	−79 (2)
N1—C7—C8—N2	55.4 (4)	O3—C23—C24'—F4'	−18.9 (12)
N2—C9—C10—N1	−57.1 (5)	O4—C23—C24'—F4'	160.3 (11)
C16—C11—C12—C13	−0.3 (5)	O3—C23—C24'—F6'	−140.4 (8)
N3—C11—C12—C13	176.4 (3)	O4—C23—C24'—F6'	38.8 (9)
C11—C12—C13—C14	1.1 (5)	C6—C1—N1—C7	−10.1 (4)
C12—C13—C14—C15	−1.1 (5)	C2—C1—N1—C7	171.1 (3)
C13—C14—C15—C16	0.3 (5)	C6—C1—N1—C10	−141.4 (3)
C14—C15—C16—C11	0.5 (6)	C2—C1—N1—C10	39.8 (4)
C12—C11—C16—C15	−0.5 (5)	C8—C7—N1—C1	171.8 (3)
N3—C11—C16—C15	−177.2 (3)	C8—C7—N1—C10	−54.8 (4)
N3—C17—C18—N4	56.6 (5)	C9—C10—N1—C1	−170.6 (3)
N4—C19—C20—N3	−55.9 (4)	C9—C10—N1—C7	55.5 (4)
O1—C21—C22—F3	−132.4 (6)	C10—C9—N2—C8	56.8 (4)
O2—C21—C22—F3	49.2 (8)	C7—C8—N2—C9	−55.8 (4)
O1—C21—C22—F1	110.7 (6)	C16—C11—N3—C20	−165.9 (3)
O2—C21—C22—F1	−67.7 (6)	C12—C11—N3—C20	17.6 (4)
O1—C21—C22—F2	−5.5 (8)	C16—C11—N3—C17	−33.3 (4)
O2—C21—C22—F2	176.1 (6)	C12—C11—N3—C17	150.2 (3)
O1—C21—C22'—F2'	47.1 (18)	C19—C20—N3—C11	−174.6 (3)
O2—C21—C22'—F2'	−131.3 (17)	C19—C20—N3—C17	51.5 (4)
O1—C21—C22'—F3'	−79.2 (9)	C18—C17—N3—C11	173.3 (3)
O2—C21—C22'—F3'	102.4 (10)	C18—C17—N3—C20	−52.1 (4)
O1—C21—C22'—F1'	170.6 (10)	C20—C19—N4—C18	59.6 (4)
O2—C21—C22'—F1'	−7.8 (12)	C17—C18—N4—C19	−60.0 (5)

### 4-Phenylpiperazin-1-ium trifluoroacetate 0.123-hydrate (IV) Hydrogen-bond geometry (Å, º)

*Cg*2 is the centroid of the C1–C6 phenyl ring.

**Table:**

*D*—H···*A*	*D*—H	H···*A*	*D*···*A*	*D*—H···*A*
N2—H21···O1^i^	0.88	1.91	2.790 (4)	174
N2—H22···O3	0.87	2.04	2.860 (4)	157
N2—H22···O4	0.87	2.47	3.164 (5)	137
N4—H41···O4^ii^	0.86	1.95	2.759 (6)	156
N4—H42···O2^iii^	0.89	1.90	2.758 (4)	164
C18—H18*A*···F5′^iii^	0.97	2.53	3.273 (18)	134
C18—H18*B*···Ow1	0.97	2.08	2.929 (15)	145
C19—H19*B*···O3^iv^	0.97	2.59	3.420 (5)	144
C20—H20*A*···F5^iv^	0.97	2.64	3.468 (8)	144
C16—H16···*Cg*2^v^	0.93	2.99	3.745 (4)	140

Symmetry codes: (i) *x*, *y*+1, *z*; (ii) *x*+1, *y*, *z*; (iii) −*x*+1, −*y*+1, −*z*; (iv) −*x*+1, −*y*+2, −*z*; (v) −*x*+1, −*y*+1, −*z*+1.
